# Characterization of mammalian Lipocalin UTRs *in silico*: Predictions for their role in post-transcriptional regulation

**DOI:** 10.1371/journal.pone.0213206

**Published:** 2019-03-06

**Authors:** Andres Mejias, Sergio Diez-Hermano, Maria D. Ganfornina, Gabriel Gutierrez, Diego Sanchez

**Affiliations:** 1 Departamento de Genetica, Universidad de Sevilla, Sevilla, Spain; 2 Instituto de Biologia y Genetica Molecular-Departamento de Bioquimica y Biologia Molecular y Fisiologia, Universidad de Valladolid-CSIC, Valladolid, Spain; 3 Departamento de Matemática Aplicada, Universidad Complutense, Madrid, Spain; University of Essex, UNITED KINGDOM

## Abstract

The Lipocalin family is a group of homologous proteins characterized by its big array of functional capabilities. As extracellular proteins, they can bind small hydrophobic ligands through a well-conserved β-barrel folding. Lipocalins evolutionary history sprawls across many different taxa and shows great divergence even within chordates. This variability is also found in their heterogeneous tissue expression pattern. Although a handful of promoter regions have been previously described, studies on UTR regulatory roles in Lipocalin gene expression are scarce. Here we report a comprehensive bioinformatic analysis showing that complex post-transcriptional regulation exists in Lipocalin genes, as suggested by the presence of alternative UTRs with substantial sequence conservation in mammals, alongside a high diversity of transcription start sites and alternative promoters. Strong selective pressure could have operated upon Lipocalins UTRs, leading to an enrichment in particular sequence motifs that limit the choice of secondary structures. Mapping these regulatory features to the expression pattern of early and late diverging Lipocalins suggests that UTRs represent an additional phylogenetic signal, which may help to uncover how functional pleiotropy originated within the Lipocalin family.

## Introduction

Lipocalins are extracellular proteins that share an ability to bind small hydrophobic ligands and a highly conserved β-barrel folding [1], though their primary sequences diverge greatly among paralogous groups [2]. Proteins in this family also show a wide functional diversity and moonlighting properties [3] that parallel their heterogeneous tissue expression patterns.

Mechanisms controlling gene expression have been studied in a handful of Lipocalins, mainly focused on their promoter regions [4,5,6,7,8]. The post-transcriptional control of gene expression exerted by the upstream and downstream untranslated regions (5’ UTR and 3’ UTR) has gained importance in recent years [9]. UTRs influence translation efficiency, mRNA molecule stability and its export outside the cell nucleus [10], to the extent that mutations in these regions are associated to severe diseases [9]. Nucleotide sequence motifs found in UTRs interact with RNA-binding proteins thanks to hairpin-like secondary structures, and non-coding RNAs like miRNAs can bind to targets in UTRs, especially in 3’ UTR [9]. Scarce information is available about UTR regulatory roles in Lipocalin gene expression and a relationship between post-transcriptional control mechanisms and the Lipocalins pleiotropic potential has not been examined.

The Lipocalin evolutionary history stands out for its vast branching across different taxa [11]. Metazoans could have inherited an ancestral prokaryotic Lipocalin gene, which after successive duplication rounds gave rise to the tens of paralogs that can be currently found in chordates. The evolutionary process followed by chordate Lipocalin genes has been studied using phylogenetic signals derived from both the gene coding sequence (CDS, namely amino acid sequence alignments) and the exon-intron architecture [12].

In this work, we analyze *in silico* the UTR regulatory regions of Lipocalins, which might represent an additional phylogenetic signal to uncover how functional diversity originated within the Lipocalin family given their aforementioned characteristics. We focus on mammalian Lipocalins because abundant information of gene orthologs is available and facilitates direct comparisons. The existence of alternative UTRs is examined, as it represents a frequent phenomenon in eukaryotic genomes that would allow a finer and more flexible gene expression control [13].

## Material and methods

### Selection and collection of 5’ and 3’ UTRs of mammalian Lipocalin sequences

Sequences from rodent and human Lipocalin orthologs were selected as representative members of the mammalian Lipocalins from the AceView database [14]. The selection was based on their position in a gene phylogeny tree [2,3,11,12] so that both early diverging (ED) and late diverging (LD) Lipocalins are represented in the study sample. We selected Lipocalins for which we found sufficient information of orthologous mammalian genes in the databases used in this work. The Lipocalin α1-microglobulin was not included in our sample because their particular gene fusion to Bikunin could uniquely affect their UTR evolutionary history.

Only transcripts with coincidence with the predicted CDS annotated in RefSeq (NCBI) were chosen. Nucleotide sequences obtained from AceView were present in ASPIcDB [15], which also allowed to include alternative transcripts. Both annotations were confirmed with NCBI RefSeq at the time of sequence selection for our catalog. When comparisons expand to species from other mammalian orders, the UTRs of the genes annotated in RefSeq were chosen.

Sequences and alignments used in this work will be available in S1–S5 Files.

### Analysis of 5’ and 3’ UTRs sequences

UTR regions were analyzed with EMBOSS Infoseq [16] in search of variables such as sequence length and G+C content. Length and G+C content of UTR Lipocalins were compared to a sample of 1000 sequences of human and rodent genes randomly chosen from UTRdb [17]. Repetitive motifs were located with Repeatmasker (A.F.A. Smit, R. Hubley & P. Green; http://repeatmasker.org). Existence of upstream initiation codons (uAUG) and their context were carried out with EMBOSS Dreg and upstream open reading frames (uORF) with EMBOSS Getorf.

Oligonucleotide analyses in search of overrepresented oligonucleotides were performed with Regulatory Sequence Analysis Tools (RSAT) (http://rsat.sb-roscoff.fr/) [18] using human and mouse background models. To predict structural motifs and estimate the minimum folding energy we used UTRscan (http://itbtools.ba.itb.cnr.it/utrscan) [17], RNAfold (http://rna.tbi.univie.ac.at//cgi-bin/RNAWebSuite/RNAfold.cgi) [19], RNAshape and RNAlocomotif (http://bibiserv2.cebitec.uni-bielefeld.de/rna) [20,21]. Synonymous and non-synonymous substitution analysis was performed with SNAP https://www.hiv.lanl.gov/content/sequence/SNAP/SNAP.html [22].

Target regions for micro RNAs (miRNA) were predicted using the PITA algorithm (https://genie.weizmann.ac.il/pubs/mir07/mir07_prediction.html) using 8 as the minimum seed size, allowing single G:U and mismatch, and using flanks to calculate site accessibility [23]. Although other miRNA prediction algorithms exist, we chose PITA due to its consideration of sequence base-pairing, free energy target accessibility and flanking sequences to test whether the existence of potential miRNA target sites is an evolutionary trait in Lipocalin diversity.

### Organization and origin of alternative 5’ UTRs

EMBOSS ESIM4 [24] was used to align alternative 5’ UTR sequences with the corresponding genomic region. AceView database annotations were used to map exon-intron organization into the alignment. 5’ UTR genomic regions were additionally examined with ExonScan [25] to predict potential exons. The presence and category of constitutive, alternative or cryptic splicing sites flanking exons were predicted with ASSP [26].

Promoter regions were identified as those annotated by the ENCODE project [27], and predicted by the NNPP algorithm [28]. We also confirmed the NNPP predictions in two Lipocalins (The ED-Lipocalin Rbp4, and the LD-Lipocalin Lcn2) with predictions of the different algorithms FPROM [29], and GPMiner [30]. FPROM predictions coincide with those NNPP of higher probability. Likewise, GPMiner predictions also show results compatible with NNPP for both Lipocalins (S1 Table). The 5’ UTR and 2 kb-upstream sequences were used for each selected Lipocalin to detect possible alternative promoters.

### UTR exon genomic conservation

Predicted exons were mapped to the genome of different mammalian orders (primates, rodents, artiodactyls and carnivores) using BLAT [31]. Retrieved sequences with percent identity >60% and presumably located in correct positions were marked as potential UTR exon orthologues. We chose the 60% identity as a stringent criterion to maximize homology, because the conservation of human and mouse orthologous sequences ranges 60–80% [32] and the ~60% conservation in the 3rd position of orthologous coding sequences. The presence of selected sequences in transcript UTRs of expression datasets was assessed using BLAST [33].

### UTR secondary structure prediction

To predict the minimal folding energy (MFE), as well as the suboptimal structures of Lipocalin UTRs, we used the RNAshape algorithm (http://bibiserv.techfak.uni-bielefeld.de/rnashapes) [34] selecting a range of free energy of +5 Kcal/mol for the suboptimal structures. Native structures show energy values closed to the MFE, and RNAshape uses 5 Kcal/mol as a default to predict alternative forms because native structures of structural RNAs show similar energy values.

We evaluated structural similarities of the predicted alternative UTR structures with RNAforester (http://bibiserv2.cebitec.uni-bielefeld.de/rnaforester) [35], and the structures were studied with PseudoViewer [36].

### Post-transcriptional regulation of Lipocalin expression

Protein abundance levels were obtained from PaxDb 4.1 (https://pax-db.org/) in human and mouse whole-integrated proteomes. Ranking and percent normalization to the overall protein abundance were estimated.

The mRNA expression levels and distribution were extracted from databases of RNA-Seq of Human tissues (Illumina Body Map; https://www.ebi.ac.uk/arrayexpress/experiments/E-MTAB-513/) and nine Mouse tissues (https://www.ebi.ac.uk/arrayexpress/experiments/E-MTAB-2801/).

## Results and discussion

### Characterization of UTRs in mammalian Lipocalins

#### Length and composition

**Fig 1:**
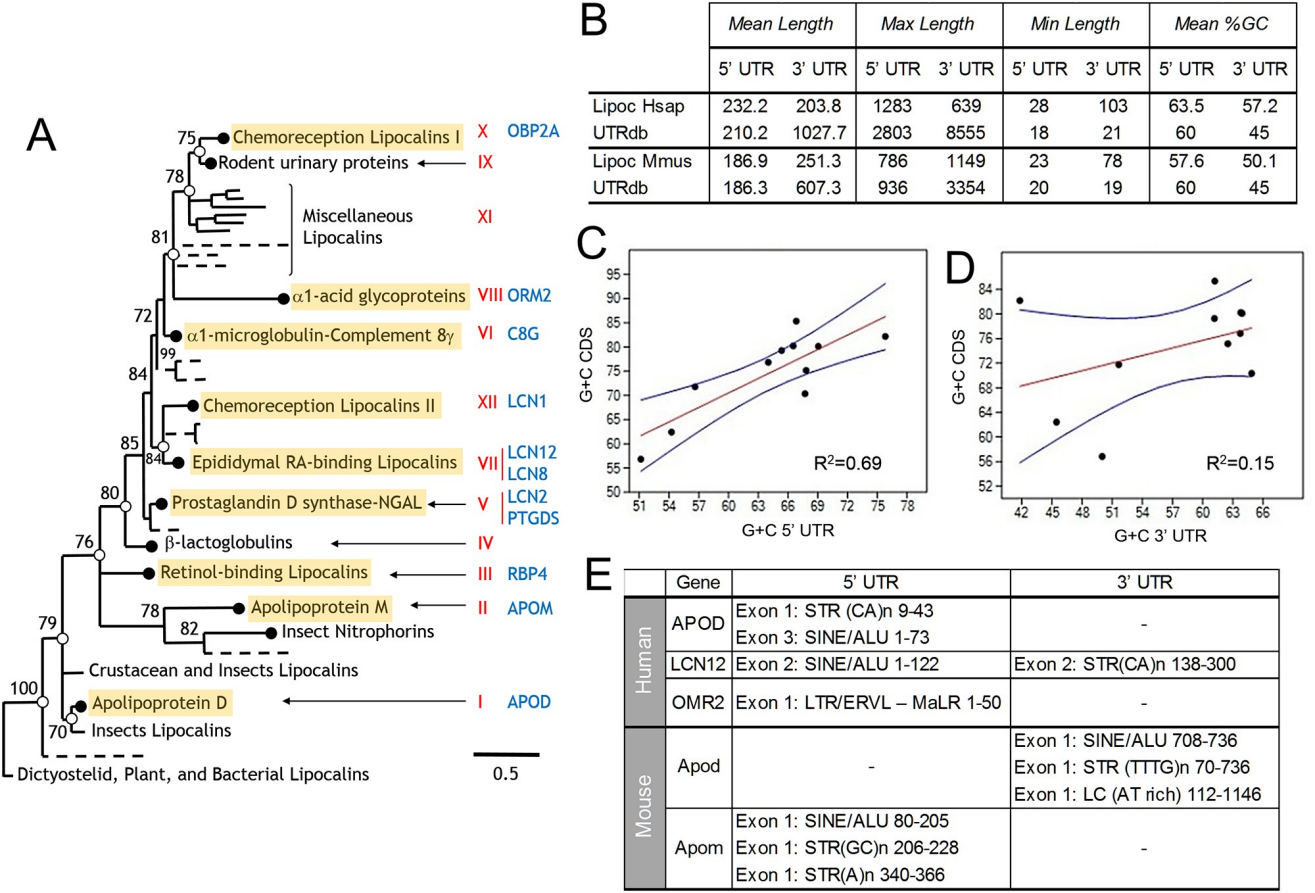
Characterization of mammalian Lipocalin UTRs. **(A)** Lipocalins selected for this study mapped on the Lipocalin family protein phylogeny. Red roman numbers indicate monophyletic clades within the family [extracted from 2]. **(B)** Average length and G+C content of human (Hsap) and mouse (Mmus) Lipocalins in comparison with average value obtained from the general UTR database for each species. **(C-D)** G+C content correlation between human Lipocalin 5’ UTRs (C) or 3’ UTRs (D) and the coding sequence (CDS) of each gene. Red lines represent the regression lines, and blue lines show the 95% confidence interval. **(E)** Repetitive elements identified in 5’ and 3’ UTRs of human and mouse Lipocalins. STR: Short Tandem Repeat; SINE: Short Interspersed Nuclear Elements; ALU: Arthrobacter luteus transposable element; ERVL: Endogenous retrovirus-related retrotransposon; LTR: Long terminal repeat; MaLR: Mammalian long terminal repeat retroposons; LC: Low complexity domains; repeating sequence or property indicated in parenthesis.

A sample of eleven human and murine Lipocalins were chosen according to their position in the family tree (Fig 1A) based on our previous phylogenetic analyses [2,3,11,12]. Early-diverging (ED) Lipocalins are represented by APOD, APOM, RBP4 and PTGDS, and Late-diverging (LD) Lipocalins by LCN2, LCN8, LCN12, LCN1, C8G, ORM2 and OBP2A. Overall, Lipocalin 5’ UTRs possess length and G+C content values similar to the global average found in the UTR database in both species, whereas Lipocalin 3’ UTRs tend to diverge from average values (Fig 1B). Mammalian 3’ UTRs are over three times longer than 5’ UTRs on average [37], a larger proportion than that of Lipocalins.

The G+C content of gene UTRs and third codon position of CDS are known to correlate [37,38], which holds true for Lipocalin 5’ UTRs (Fig 1C). However, no significant correlation was found for Lipocalin 3’ UTRs (Fig 1D), with a G+C content higher than expected for their length [39]. These results suggest that Lipocalin 3’ UTRs G+C content does not properly reflect the features of their genomic context and support the idea that mammalian Lipocalin 3’ UTRs have adapted along their evolutionary history to specific gene expression regulatory needs.

#### Repetitive elements

Some eukaryotic UTRs appear enriched in repetitive elements (STR, LINE, SINE, LTR), mostly found in the 3’ UTR, with frequencies associated to functional roles [38]. Repetitive motifs are found in some human and murine Lipocalin UTRs (Fig 1E). The most common elements are SINE/ALU and STR, in agreement with the expected mammalian UTRs [38]. There are clear differences in the 5’ and 3’ distribution of repetitive elements between human and mouse orthologues for some Lipocalins, suggesting that their contribution to regulate Lipocalin gene expression is species-specific. Since some repetitive elements span over a hundred nucleotides (Fig 1E), and they even give origin to new alternative exons, they could likely play a role in generating UTR variability during Lipocalin evolution.

#### Alternative UTRs

**Fig 2:**
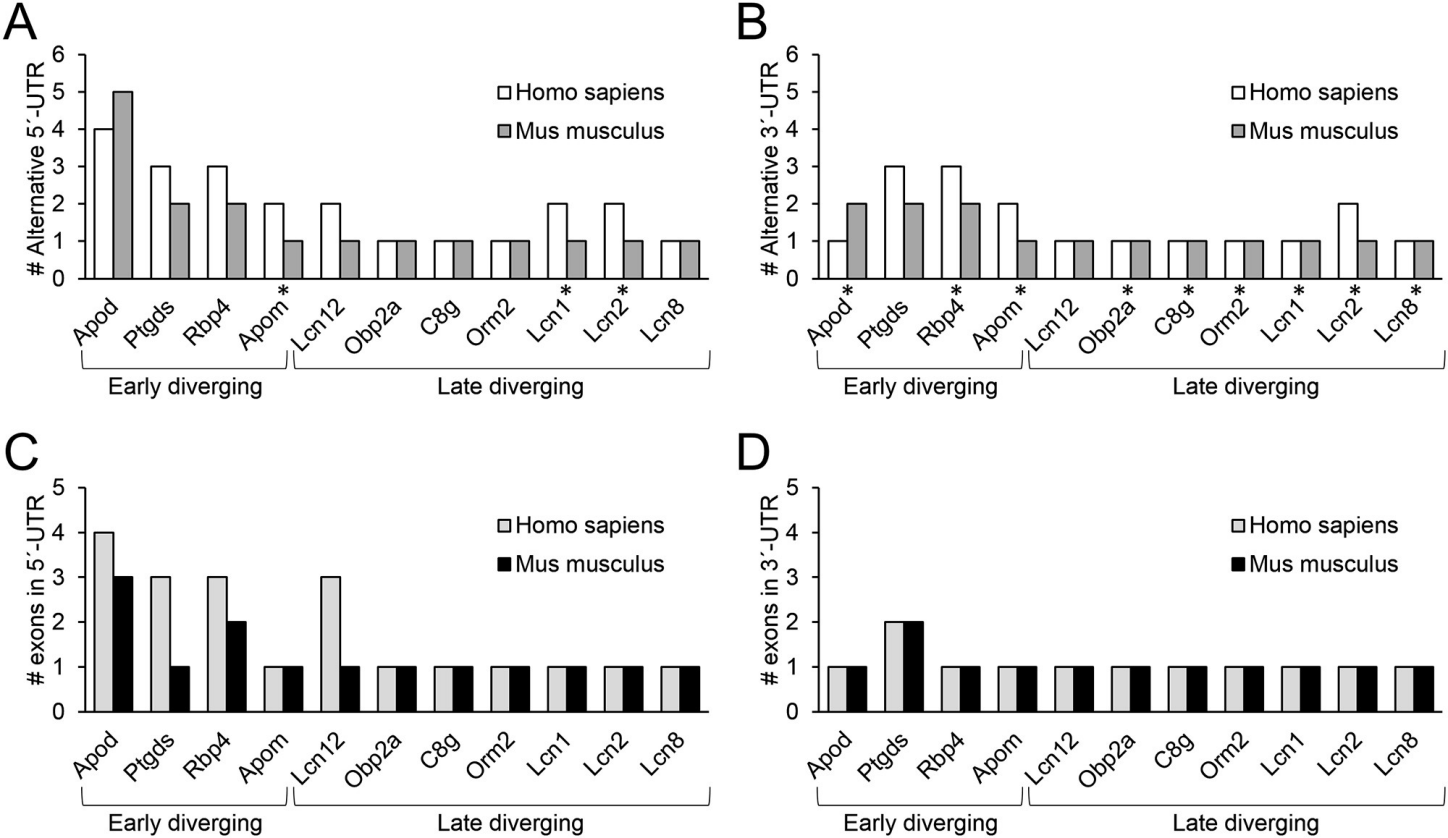
Diversity in intron-exon structure of human and murine Lipocalin UTRs. **(A-B)** Number of alternative 5’ UTRs (A) or 3’ UTRs (B) in early and late diverging Lipocalins. Single exon alternative forms are pointed by asterisks. **(C-D)** Maximum number of exons present in the 5’ UTRs (C) or 3’ UTRs (D) of selected Lipocalins.

Lipocalin UTRs display sequence variation, and many genes selected for this work show alternative 5’ UTRs both in mouse and human (Fig 2A). Furthermore, we find a tendency to present high number of alternative 5’ UTRs in ED-Lipocalins such as APOD, PTGDS and RBP4. In contrast, alternative 3’ UTRs (Fig 2B) are not so common in Lipocalins, but also appear to be more frequent in ED-genes. In general, human Lipocalins tend to have more alternative UTRs than murine ones.

Considering the mechanisms underlying alternative UTR forms, we compiled the number of UTR exons found in Lipocalins (Fig 2C and 2D). RNA Alternative splicing explains the origin of alternative forms in most cases. However, among Lipocalins with a single 5’ UTR exon, human APOM, LCN1 and LCN2 still possess alternative forms (asterisks in Fig 1A), suggesting the existence of alternative transcription start sites (see below).

In relation to 3’ UTRs, the two exons detected in human and murine PTGDS (Fig 2D) support a splicing mechanism for the predicted alternative forms. All other Lipocalins in the set studied have single exon 3’ UTRs (Fig 2D). However, some of them (APOD, RBP4, APOM and LCN2) bear alternative forms (Fig 2B) that can be originated by variable cleavage at different polyadenylation sites.

### Evolution of 5’ and 3’ UTRs in mammalian Lipocalins

#### 5’ UTR evolution

**Fig 3:**
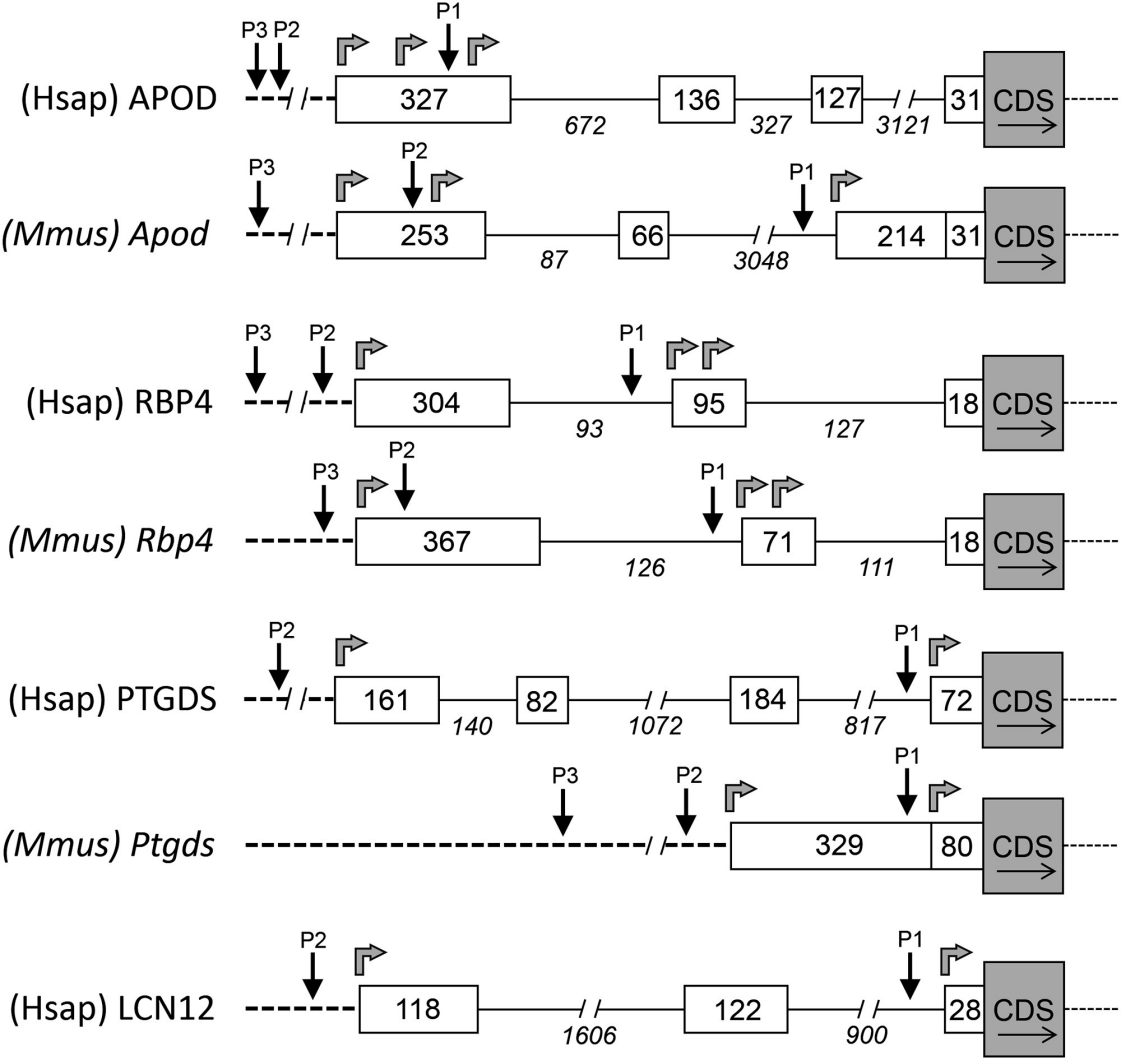
Architecture of genomic region of human and mouse Lipocalins with multiexonic 5’UTRs. Exon-intron structure for human (Hsap) and murine (Mmus) Lipocalin genes upstream of their CDS. Black arrows point to predicted alternative promoters (P). Gray arrows indicate alternative transcription initiation sites.

A set of features found in the different alternative 5’ UTRs of human and mouse Lipocalins are compiled in Table 1, where each alternative form is denoted by a letter suffix. To learn about the evolution of mammalian Lipocalin 5’ UTRs, we first analyzed the genomic architecture of exons/introns for human and murine genes that show alternative and multiexonic 5’ UTRs in both species. Fig 3 displays a schematic view of the genomic regions of these 5’ UTRs. The NNPP algorithm and the ENCODE project predict alternative gene promoters that are coherent with several transcription start sites in some Lipocalins such as human and murine APOD, RBP4, PTGDS, and human LCN12. In Lipocalins not showing 5’ UTR variability (Fig 2A), ExonScan and ENCODE detected neither additional upstream exons nor candidate promoter regions. Interestingly, the ED-Lipocalins APOD and RBP4 show clear similarities between murine and human exon/intron structure (Fig 3), as well as alternative gene promoters and transcription start sites. However, PTGDS shows species-specific 5’ UTR exon-intron structures, quite dissimilar between human and mouse genes.

**Table 1:** Features of alternative 5’ UTR of human and murine Lipocalins.

	Lipocalin_Alt 5' UTR	Length		% G+C		MFE (Kcal/mol)		# uAUG		# uORF		uORF translation efficiency		uORF sequence context		Overrepresented oligos		CART Class	
		*Human*	*Mouse*	*Human*	*Mouse*	*Human*	*Mouse*	*Human*	*Mouse*	*Human*	*Mouse*	*Human*	*Mouse*	*Human*	*Mouse*	*Human*	*Mouse*	*Human*	*Mouse*
EDL	ApoD_a	**361**	140	51.25	50.71	-102.2	-42	4		3		O3		O3		1		I	I
	ApoD_b	232	224	53.88	49.55	-87.5	-69.1	1	1	1	1	O1	O1	O1	O1	1	1	I	I
	ApoD_c	135	358	47.41	51.4	-35.5	-126.94	2	2	1	2	O1	O2	O1	O2	1	1	I	I
	ApoD_d	190	**281**	52.11	53.74	-60.8	-103.24	3	2	2	2	O1/W1	O2	O2	O2	1	2	I	I
	ApoD_e		214		59.35		-74.4		2		1		W1		O1				I
	Rbp4_a		**385**		62.08		-148.1		4				O4	O3/W1			1		I
	Rbp4_b	322		72.67		-179.5		1		1		W1		O1		4		I	
	Rbp4_c		61		77.05		-18.7	1		1		W1		O1					III
	Rbp4_d	**72**	89	77.78	77.53	-28.7	-37.7										1	III	I
	Rbp4_d(2)	113		76.99		-46.4												I	
	Ptgds_c	**72**	**80**	69.44	56.25	-20.5	-20.2											III	III
	Ptgds_d		329		58.36		-149.23		2		2		O2		O1/W1		1		I
	Ptgds_g	458		65.5		-203.8		4		4		O4		O2/W2				I	
	Ptgds_j	1283		65.55		-644.51		11		5		O5		O4/W1		4		I	
	ApoM_a		**781**		48.27		-240.6		14		8		O8		O2/W6		1		I
	ApoM_d	496		49.6		-161.77		6		5		O5		O2/W3		1		I	
	ApoM_e	**73**		58.9		-17.4												III	
LDL	C8G_a	**75**		64		-21.5												III	
	C8G_b		501		53.69		-188.12		6		6		O6		O1/W4				I
	C8G_c		**275**		53.82		-104.22		4		4		O3/W1		O1/W2				I
	Obp2A_b	**42**		69.05		-15.2												III	
	Obp2A_a		**53**		62.26	-17.5													III
	Orm2_a		**41**		53.66		-3.4												III
	Orm2_b	**189**		56.61		-76.5										1		I	
	Lcn1(Vegp1)_a		**55**		54.55		-18.1												III
	Lcn1_b	**154**		63.64		-63.4										1		I	
	Lcn1_h	49		69.39		-9												III	
	Lcn2_b	**72**	**54**	67.71	53.7	-13	-7.3											III	III
	Lcn2_b(2)	96		67.71		-25.3										2		III	
	Lcn8_a		**23**		65.22		-3.6												III
	Lcn8_e	**348**		67.82		-166.1		3		3		O3		O1/W2		2		I	
	Lcn12_a		**55**		69.09		-30.3												III
	Lcn12_c	**248**		59.27		-99.8		1		1		O1		O1		1		I	
	Lcn12_c(2)	28		71.43		-8.1												III	

Features for each 5’ UTR sequence of the Lipocalin sample set under study. The sequence length highlighted in bold marks the RefSeq 5’ UTR for each organism. MFE: Minimal folding energy; uAUG: upstream initiation codon; uORF: upstream open reading frame. uORF translation efficiency relates to the distance of uORFs to the 5´cap of the mRNA, and it is classified in two categories: Optimal (O: >19 nucleotides), or Weak (W: <12 nucleotides). Within those categories, indices 1 to 8 are assigned according to the number of uORFs present. uORF sequence context is related to the consensus sequence in positions -3 and +4 surrounding the start (AUG) codon. As above, they are classified as optimal (O), or weak (W) and indexed depending on the number of uORFs present. CART class: I: Low translation; III: efficient translation. EDL: Early diverging Lipocalins. LDL: Late diverging Lipocalins.

**Fig 4:**
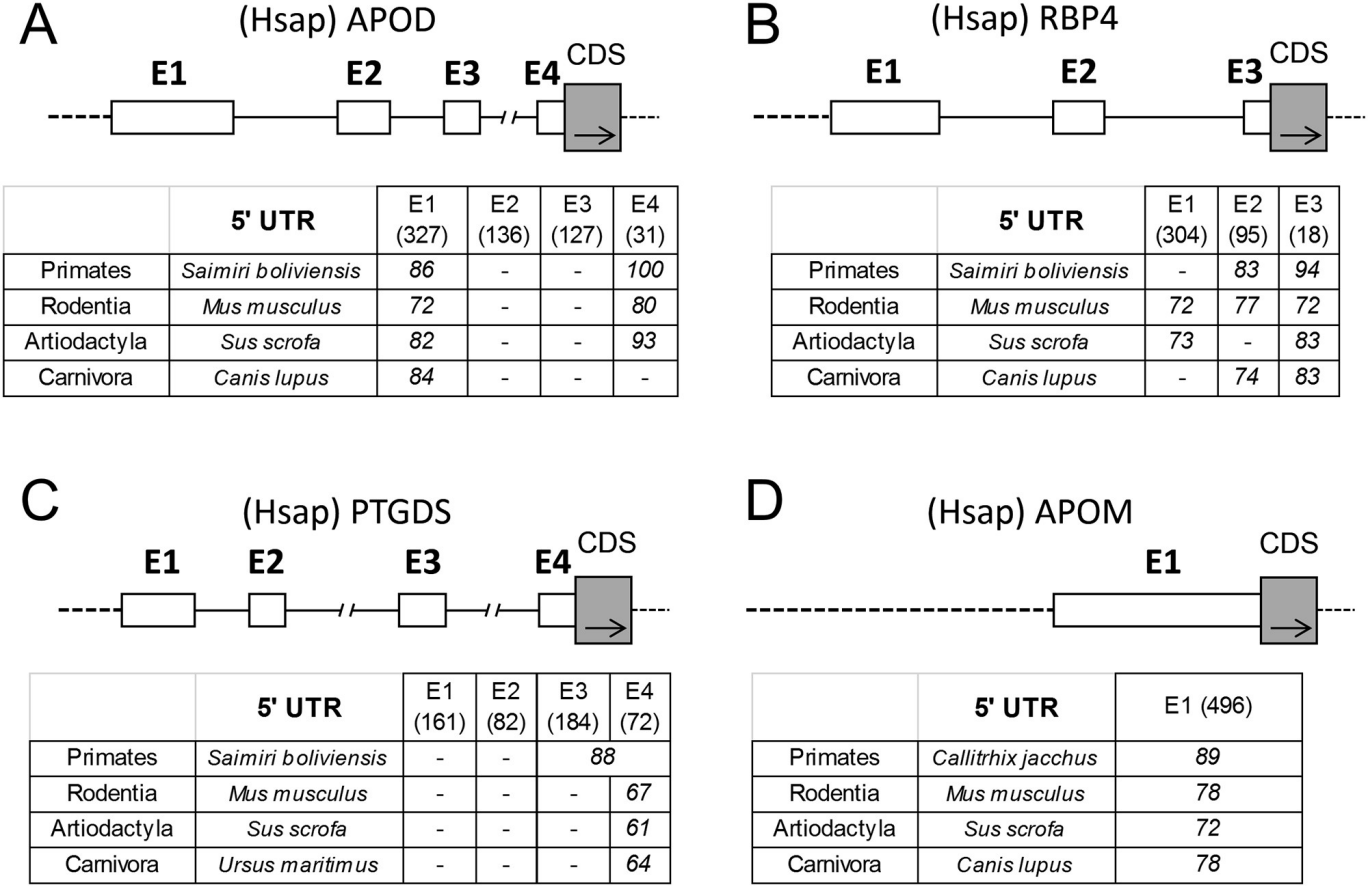
Sequence similarity of orthologous 5’ UTR exons. Sequence similarity between human 5’ UTR exons of APOD (A), RBP4 (B), PTGDS (C) and APOM (D) versus the orthologous ones from selected species of different mammalian orders. The complete exon-intron structure of 5’ UTR for each human Lipocalin is shown for reference. Percent identity (≥60% identity) obtained from pairwise alignments are shown. (-): Lack of homologous exon.

We then calculated the degree of similarity between exons of human Lipocalin 5’ UTRs *versus* selected species of different mammalian orders (primates, rodentia, artiodactyla and carnivora) (Fig 4). Orthologous pairs of exons were compared. Pairwise alignments reveal that some of the human 5’ UTRs exons of APOD, RBP4 and PTGDS (Fig 4A–4C) show significant sequence similarity (>60% identity), indicating conservation along the mammalian orders studied. However, other exons in the same UTRs show no significant similarity with other species, which could be considered hominidae synapomorphies. As for APOM (Fig 4D), its unique 5’ UTR exon also shows significant similarity (72–89% identity) with those of other mammalian orders. However, the single 5’ UTR exons of LD-Lipocalins display no significant similarity with other mammals.

We also compared average percent identities of orthologous 5’ UTRs exons with those obtained when analyzing the corresponding coding sequences (CDS) in the mammalian orders shown in Fig 4. Table 2 shows that values of percent identity in 5’ UTRs are similar to those for the third position of CDS codons in ED-Lipocalins, but much lower in LD-Lipocalins. This result indicates the existence of a strong selective pressure operating in the 5’ UTRs of early diverging mammalian Lipocalins.

**Table 2:** 5’ UTR—CDS divergence comparisons.

		Identity (%) CDS 1^st^ & 2^nd^ nuc	Identity (%) CDS 3^rd^ nuc	Identity (%) 5' UTR
EDL	APOD	86.33	70.9	78.2
	RBP4	86.62	75.3	71.7
	PTGDS	84.4	71.8	65.7
	APOM	90.78	75.11	72.1
LDL	LCN12	71	60.2	45.6
	LCN2	75.2	66.7	47.1
	LCN8	80.6	63.3	37

Sequence similarity (% identity) of orthologous 5’ UTRs is compared with % identity in the different codon positions of their corresponding coding sequences (CDS). Data obtained from the mammalian orders used in Fig 4. EDL: Early diverging Lipocalins. LDL: Late diverging Lipocalins.

**Fig 5:**
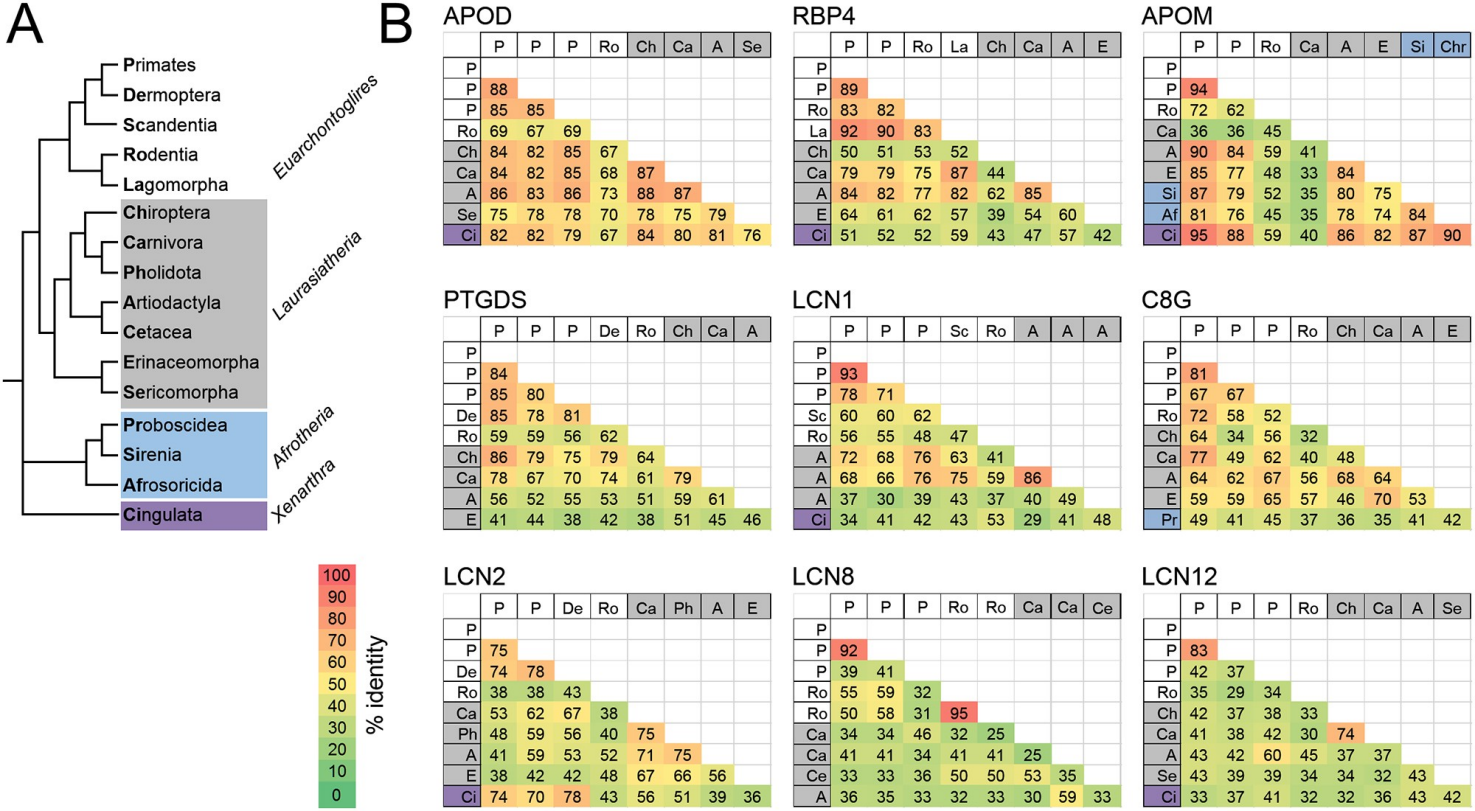
Distance matrix analysis of Lipocalin 5’ UTRs along mammalian evolution. **(A)** Cladogram of the set of 16 mammalian orders, belonging to three Eutherian taxonomic ranks, used for the comparison of RefSeq 5’ UTRs of Lipocalins. Color code is used in A and B to indicate evolutionary depth. **(B)** Distance matrices obtained from multiple sequence alignments (MSA) are shown color-coded. Number represent sequence similarity (% identity) in different mammalian orders of the RefSeq 5’ UTRs of nine out of the eleven Lipocalins studied in this work. Missing sequences of OBP2A and ORM2 in several orders precluded an analysis with sufficient evolutionary depth for these Lipocalins.

Considering the RefSeq 5’ UTRs of the Lipocalins studied in this work (bold letters in Table 1), we performed a multiple sequence alignment (MSA) in a set of 16 mammalian orders belonging to three Eutherian taxonomic ranks that cover 120 My of mammalian evolution. The result of the pairwise percent identities (distance matrices) are graphically shown in Fig 5. The pattern supports that ED-Lipocalins display a strong sequence conservation of their 5’ UTR throughout mammalian evolution, while LD-Lipocalins show high variability in their sequence even among species of the same order.

#### 3’ UTR evolution

Overall, the genomic architecture of Lipocalin 3’ UTRs is simpler than that of 5’ UTRs (Fig 2). Only PTGDS present a single intron. Lipocalin 3’ UTRs seem fairly conserved within primates, with identities in the range of 88–96%, and a fair degree of conservation (>60%) in most other cases (Table 3). However, the lack of complete 3’ UTR sequences in the databases for some Lipocalins precluded a broad analysis. With the data available so far, these results provide evidence for an important regulatory function of 3’ UTRs in Lipocalin expression.

**Table 3:** Sequence similarity 3’ UTRs.

3' UTR	APOD	RBP4	PTGDS	APOM	LCN8
Primates	88	96	85	92	95
Rodentia	68	73		67	70
Artiodactyla	80	78	60	79	67
Carnivora	76	74	66	88	60

Average sequence similarity (% identity) of orthologous RefSeq 3’UTRs for different mammalian orders. Only significant similarities (≥60%) are shown.

### Properties of mammalian Lipocalin 5’ UTR sequences influencing regulatory complexity of protein expression

Because of the different prevalence of alternative forms and the differences in sequence conservation of Lipocalin 5’ UTRs depending of their evolutionary history, variations are also expected in the regulatory elements present in these gene regions.

Length, G+C content, several sequence motifs and secondary structure are 5’ UTR features that could play an important role in gene expression regulation. Short 5’ UTRs, with low G+C content and low degree of secondary structure allow efficient translation, while the contrary holds for genes showing low translation levels [40,41]. Similarly, the existence of upstream initiation codons (uAUG) and upstream open reading frames (uORF) is generally assumed to involve a negative regulation of translation [42,43,44], whose strength relies on properties such as an appropriate sequence context [45], enough distance (>19 nucleotides) to the 5’ cap, the presence of multiple uORFS, and their evolutionary conservation.

Overrepresented sequence elements in 5’ UTRs can be considered regulatory motifs. A low incidence rate categorize 6–8 nucleotide oligonucleotides as significant. Moreover, an overlap of different oligonucleotides and their evolutionary conservation favor their regulatory role [46].

We searched for the features above in our set of human and murine Lipocalin genes 5’ UTRs, and these data were used to categorize the translation efficiency of our UTRs according to the classification and regression tree (CART) method [47]. The overall results are compiled in Table 1.

Significantly overrepresented oligonucleotides in human Lipocalins are CTGGCA and TGCCAG (Observed: 16; Expected: 2.77; Significance Index: 3.77), CCACCC (17; 4.15; 2.13) and CAGGGCC (9; 1.18; 1.17). Two significant oligonucleotides found in mouse Lipocalins [CTGGGCA (6; 0.64; 0.04) and CCACCC (11; 2.54; 0.54)] are also conserved in human Lipocalin 5’ UTRs. However, these oligonucleotides do not correspond to any known 5’ UTR motif.

**Fig 6:**
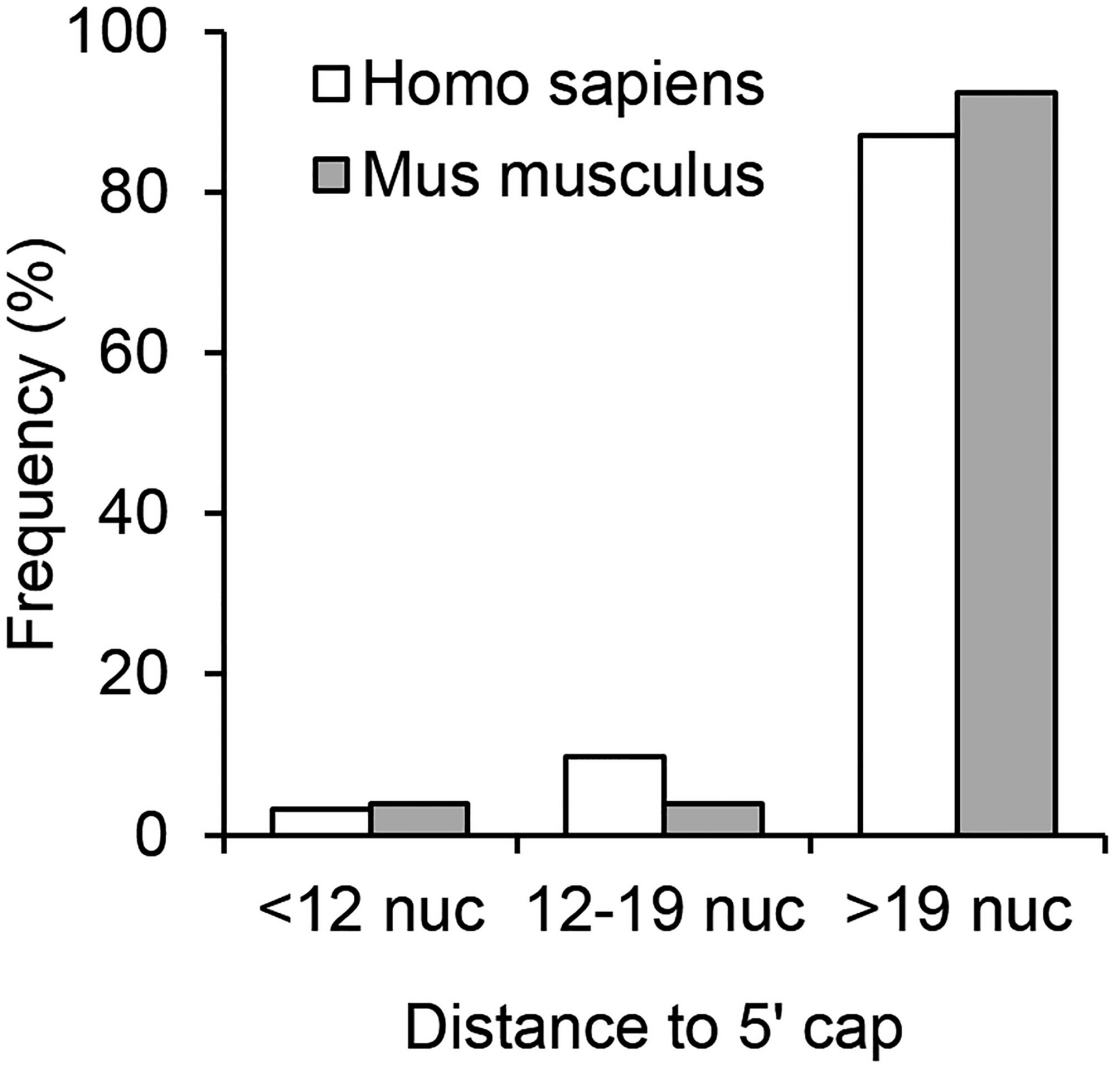
Translation efficiency predictions for human and mouse Lipocalins. Predictions are based in the frequency distribution of distances between the 5’ cap and each uORF present in Lipocalins 5’ UTRs.

We also found that human and murine Lipocalins uAUG/uORFs are abundant in other species, and many of them show an optimal/adequate context for translation (Table 1). Translation inhibition of uORFs was also predicted by measuring distances between the 5’ cap and each Lipocalin uORF (Fig 6). Together these results suggest that translated uORFs are common and efficient in Lipocalins, mainly in ED-genes (Table 1). Moreover, some Lipocalin 5’ UTR variants bearing uORFs show significant sequence conservation in several mammalian orders. Particularly, two uORFs of human APOD_a and APOM_d variants and its orthologous sequences show Ka/Ks values above one (1.587 for APOD and 1.309 for APOM) which suggests a positive selection for the peptides putatively translated from those uORFs.

Finally, the features above contributed to categorize translation efficiency as CART Class I genes (low translation), more abundant in ED-Lipocalins such as APOD and RBP4, and those with efficient translation (Class III) that correspond to LD-Lipocalins (Table 1).

In summary, more variation in terms of alternative 5’ UTRs, more sequence conservation found across evolutionarily divergent mammalian orders, as well as sequence motifs compatible with a stringent translational control, suggest that ED-Lipocalins amply present in chordates are limitedly translated.

### Properties of mammalian Lipocalin 3’ UTR sequences influencing regulatory complexity of protein expression

The sequence conservation observed in Lipocalin 3’ UTRs led us to explore whether some known regulatory features of this gene region could underlie the functional evolutionary diversity of the Lipocalin gene family.

Polyadenylation signals (PAS) are involved in mRNA cytoplasmic export and stability [48]. We analyzed the number, position, type (canonical vs. non-canonical) of PAS of human and murine Lipocalin 3’ UTRs and estimated their polyadenylation efficiency [49,50].

Table 4 shows that ED-Lipocalins APOD, RBP and PTGDS (both in human and mouse) bear long 3’ UTRs with more alternative forms. Longer variants with multiple polyadenylation sites (PAS) are predicted to have potentially complex regulation, depending on the efficiency of their PAS. In contrast, LD-Lipocalins show short 3’ UTRs with single PAS that suggests less complexity in their translation regulation.

**Table 4:** Features of alternative 3’ UTR of human and murine Lipocalins.

3'UTR		Lipocalin_Alt 3' UTR	Length	PAS position	PAS type (1)	PAS Efficiency (2)	Accessible miRNA targets (3)	Very accessible miRNA targets (4)
***Hs***	EDL	APOD_a,b,c	198	153	C	**VE**	23	13
				68	NC*	LE		
		RBP4_b	388	211	NC	LE	70	59
				360	NC	**E**		
				112	NC*	LE		
				130	NC*	LE		
		RBP4_c	186	112	NC*	LE		
				130	NC*	(LE)		
		PTGDS_c	214	191	C	VE	14	0
		PTGDS_g	178	159	C	VE	14	0
		PTGDS_j	639	142	C	LE	33	0
				510	NC*	LE		
				621	NC*	**E**		
		APOM_d,e	121	97	C	VE		
	LDL	C8G_a	193	175	NC	E		
		OBP2A_b	133	114	C	VE	61	0
		ORM2_b	122	94	C	VE		
		LCN1_b,h	185	166	NC*	E	50	20
		LCN2_b	153	130	C	VE	9	0
		LCN2_b(2)	334	315	C	**VE**	28	8
		LCN8_e	112	95	C	VE	7	1
		LCN12_c,c(2)	103	78	C	VE	30	9
***Mm***	EDL	Apod_a,b,d	223	203	C	(LE)	1	1
		Apod_c	1149	203	C	LE	7	2
				672	NC	LE		
				1128	NC	**E**		
		Rbp4_a,d	252	114	NC	LE	2	0
				225	NC	**E**		
		Rbp4_c	128	114	NC	(LE)	1	0
		Ptgds_d	159	139	C	**VE**	4	0
				135	NC*	E		
		Ptgds_e	614	594	C	**VE**	8	4
				590	NC*	E		
		Apom_a	117	89	C	VE	3	2
	LDL	C8g_b,c,d	154	136	NC	E	2	0
		Obp2A_a	164	145	C	VE	10	6
		Orm2_a	113	84	C	VE	4	0
		Lcn1(Vegp1)_a	164	146	NC*	E	15	5
		Lcn2_b	237	212	C	**VE**	8	5
				216	NC*	E		
		Lcn8_a	107	85	C	VE	7	6
		Lcn12_a	78	55	C	VE	7	5

(1) C: Canonical polyadenylation sites [AAUAAA]; NC: Non-canonical [AUUAAA]; NC*: Other less frequent types. (2) Efficiency of polyadenylation sites. VE: Very efficient; E: Efficient; LE: Low efficiency. Accessibility of miRNAs classified as accessible targets when ΔΔG < -10 (3) and as very accessible targets when ΔΔG < -10 and ΔGopen > -10 Kcal/mol (4). EDL: Early diverging Lipocalins. LDL: Late diverging Lipocalins. Hs: Homo sapiens. Mm: Mus musculus.

3’ UTRs are a common target for miRNAs, well-known regulators of gene expression [9]. We evaluated the miRNA accessibility of 3’ UTRs (Table 4), and found that human Lipocalins show more miRNA potential targets than those in the mouse, suggesting a stronger role of 3’ UTR miRNA in gene regulation of primate Lipocalins. A different strategy to assess the biological relevance of the predicted miRNA targets is to compare them among different vertebrate species. Table 5 shows a list of potential miRNA targets in human and mouse Lipocalins. Several miRNAs show 3’ UTR targets in different human Lipocalins, and miR-125a-3p is the only common miRNA predicted for an orthologous Lipocalin (Obp2a) in mouse and human.

**Table 5:** Human and mouse predicted miRNAs targets in the 3’ UTR of Lipocalins.

miRNA		Lipocalin_Alt 3' UTR	miRNA
***Hs***	EDL	APOD_a	hsa-miR-185
			hsa-miR-202
		RBP4_b	hsa-miR-125a-3p
			hsa-miR-127-3p
			hsa-miR-134
			hsa-miR-146a
			hsa-miR-185
			hsa-miR-296-3p
			hsa-miR-324-5p
			hsa-miR-363
	LDL	OBP2a_b	hsa-miR-125a-3p
		LCN1_b	hsa-miR-24
			hsa-miR-296-3p
		LCN2_b2	hsa-miR-296-3p
		LCN12_c	hsa-miR-330-5p
***Mm***	EDL	Apod_a	mmu-miR-383
		Ptgds_e	mmu-miR-202-3p
		Apom_a	mmu-miR-124
	LDL	Obp2a_a	mmu-miR-125a-3p
			mmu-miR-491
		Lcn1_a	mmu-miR-296-3p
		Lcn8_a	mmu-miR-503
			mmu-miR-214
		Lcn12_a	mmu-let-7b
			mmu-miR-449a
			mmu-miR-449b
			mmu-miR-34a

EDL: Early diverging Lipocalins. LDL: Late diverging Lipocalins. Hs: Homo sapiens. Mm: Mus musculus.

In the past few years, a number of miRNA have been found to alter experimentally the expression of some Lipocalins. miRNAs 299-3p, 423-3p and 490-3p were associated to ApoD expression in rat [51]; miRNAs 18b-5p, 19b-3p, 99a-5p, 100-5p, 145-5p, 214-3p and 138 alter Lcn2 expression [52,53], and miRNA 573 affects ApoM expression [54]. Some of these miRNAs were detected by the PITA algorithm [23], but they were below the ΔΔG threshold of -10 Kcal/mol to be considered accessible.

### Properties of mammalian Lipocalin 5’ and 3’ UTR secondary structures influencing regulatory complexity of protein expression

The secondary structure of 5’ and 3’ UTRs are known to be a key factor for their regulatory function in gene expression [13,38]. Among the possible folds of a given UTR, the native structure not always represents the one with a minimal folding energy (MFE) [34,55]. Moreover, structural RNAs show a more reduced repertoire of potential secondary structures than those of non-structural RNAs [34].

**Fig 7:**
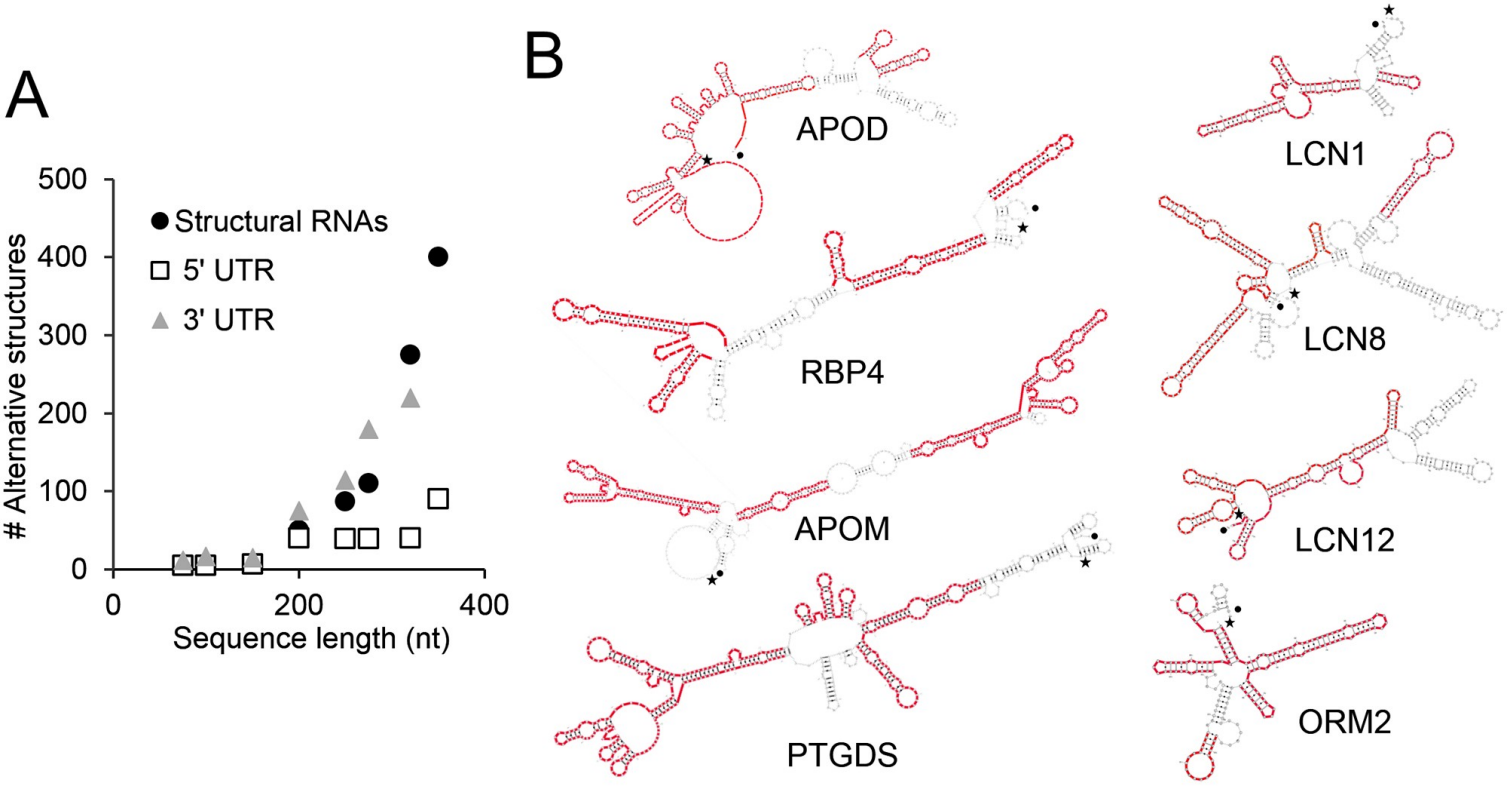
Secondary structure prediction of mammalian Lipocalin UTRs. **(A)** Comparison of the number of alternative UTR secondary structures of Lipocalins with those of structural RNAs of similar length compiled in the Rfam database. **(B)** Secondary structure prediction of 5’ UTRs of human Lipocalins. The structure with minimal folding energy (MFE) is shown for each Lipocalin. The elements shown in red represent regions showing similarity to the MFE structure in at least 60% of the suboptimal (± 5 Kcal/mol) structures. 5’ end is denoted by a star and 3’end by a dot.

Therefore, we believe it is very important to study the predicted catalogue of secondary structures of the Lipocalin UTRs in order to make informative hypotheses about their regulatory role. We analyzed the MFE and suboptimal (±5 Kcal/mol) structures of the 5’ and 3’ UTRs of our selected human and mouse Lipocalins predicted by the RNAshape algorithm (see Methods). We first compared the number of alternative UTR secondary structures of Lipocalins with those of structural RNAs (tRNAs and rRNAs) of similar length present in the Rfam database. The number of alternative secondary structures grow exponentially with the sequence length of structural RNAs (Fig 7A), and a similar relationship found in 3’ UTR Lipocalins. However, the average number of alternative secondary structures of Lipocalin 5’ UTR is significantly lower in sequences over 150 nucleotides length.

Moreover, we assessed the degree of similarity among human 5’ UTR alternative structures (over 150 nucleotides) through alignments with RNAforester (see Methods) and found slight differences between MFE and suboptimal structures (Fig 7B).

A restricted range of secondary structures suggests a high conservation of functional elements, and highlights the relevant role of 5’ UTR in Lipocalin gene regulation.

### UTR properties and post-transcriptional regulation of Lipocalin expression

**Fig 8:**
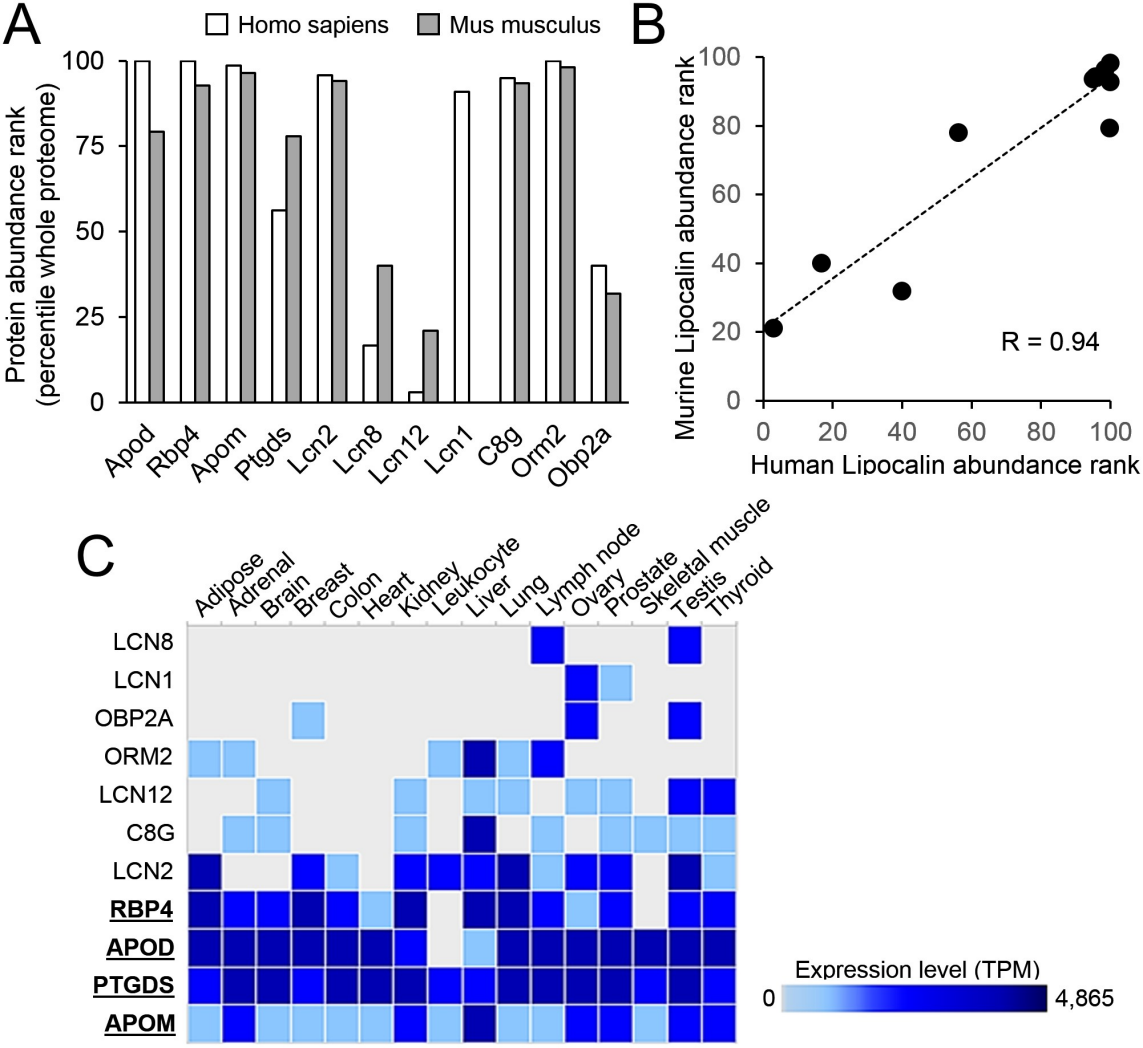
Expression level differences of Lipocalins. **(A)** Abundance levels of human and murine Lipocalins, expressed in normalized parts per million (ppm) and retrieved from PaxDb 4.1 (https://pax-db.org/), were ranked and normalized to the whole-integrated proteome. **(B)** Positive correlation of whole-organism protein abundance levels of human and mouse Lipocalins. **(C)** RNA-Seq of Human tissues (Illumina Body Map; https://www.ebi.ac.uk/arrayexpress/experiments/E-MTAB-513/). Expression levels (in transcripts per million; TPM) in different tissues show ED-Lipocalins (underlined) with a broad expression pattern, and LD-Lipocalins with a more restricted expression to certain tissues.

An apparent contrast in mRNA regulatory stringency led us to consider whether evolutionary divergence might underlie actual differences in translation efficiencies. This idea was tested by assaying protein abundance in the PaxDb 4.1 (https://pax-db.org/) for our Lipocalin set in human and mouse whole-integrated proteomes. Following ranking and percent normalization to the overall protein abundance, a general finding is that Lipocalins show high protein abundance levels in mammals (Fig 8A). These results can be explained by a substantial production of Lipocalin mRNAs that would ensure adequate protein levels despite a stringent post-transcriptional regulation. Also, a positive correlation is evident among orthologous Lipocalins (Fig 8B), in agreement with overall results when comparing human and mouse proteomes [37]. High protein levels are clear for ED-Lipocalins in mouse and human proteomes (Fig 8A), while only immune system-related acute phase LD-Lipocalins Lcn2, C8g and Orm2 show high abundance. The remaining LD-Lipocalins show scarce or even unnoticeable protein levels.

In contrast, an analysis of RNA-Seq of Human tissues (Illumina Body Map; https://www.ebi.ac.uk/arrayexpress/experiments/E-MTAB-513/) show that ED-Lipocalin transcripts are broadly present in human tissues (Fig 8C; underlined genes), while LD-Lipocalins appear more restricted to certain tissues. Similar results are obtained in a RNA-seq study (https://www.ebi.ac.uk/arrayexpress/experiments/E-MTAB-2801/) of nine mouse tissues (not shown). ED-Lipocalins broad distribution across many different tissues possibly reflects evolutionary traits that result in an increased variability and tight regulation, as suggested by alternative splicing being more common in UTR regions than in their CDS. A complex translational regulation might be responsible for a given ED-Lipocalin mRNA to be differentially expressed in diverse cellular contexts.

On the contrary, LD-Lipocalin genes display UTRs less constricted by selective pressure, with more divergent sequences across orthologs and sequence motifs usually associated with an efficient translation, alongside simpler post-transcriptional regulation mechanisms. This contrasts to their relatively low levels of protein abundance, but a plausible explanation is their tissue-specific expression pattern, which could have led to a lesser need of innovative post-transcriptional regulatory solutions.

Overall, there is an apparent “evolutionary distance/complexity” trade-off in Lipocalin gene UTR-dependent expression regulation, with ED-Lipocalins displaying tight translational regulatory mechanisms under high selective pressure, and LD-Lipocalins having tissue expression patterns loosely regulated at the post-transcriptional level.

## Conclusions

The results of our *in silico* study point to mammalian Lipocalins as a group of paralogous genes, heterogeneous in the context of expression regulation, with UTRs playing a critical role. A strong selective pressure operating upon UTRs (mainly 5’ UTR), reflecting a relevant and complex regulation of translation, is suggested by: 1) the presence of alternative UTRs accompanied by a predicted diversity of transcription start sites and alternative promoters; 2) a fair sequence conservation in different mammalian orders; 3) the existence of particular sequence motifs and other regulatory features; 4) a limited choice of secondary structures.

This is especially clear in some Lipocalins present early in vertebrate evolution that we have called ED-Lipocalins. These genes show UTR features compatible with complex regulatory mechanisms apparently motivated by the need to accommodate gene expression levels to many different cellular environments, as shown by their high abundance and ubiquitous presence in human and mouse tissues. The opposite seems to occur for LD-Lipocalins, which presumably reflects their role as functional specialists that originated as niche solutions to concrete physiological needs.

Overall, there is an apparent “evolutionary distance/complexity” trade-off in Lipocalin gene UTR-dependent expression regulation, with ED-Lipocalins displaying tight translational regulatory mechanisms under high selective pressure, and LD-Lipocalins having tissue expression patterns loosely regulated at the post-transcriptional level.

## Supporting information

S1 File Sequences used in Fig 3. (ZIP)

S2 File Sequences and alignments used in Fig 5. (ZIP)

S3 File Sequences used in Fig 1 and Tables 1, 4 and 5. (ZIP)

S4 File Sequences and alignments used in Table 3. (ZIP)

S5 File Sequences used in Fig 4. (ZIP)

S1 Table Promoter prediction comparisons. (DOCX)
