# Supplementary material for: Characterization of mammalian Lipocalin UTRs in silico: Predictions for their role in post-transcriptional regulation
Source: PLoS One. 2019 Mar 6;14(3):e0213206. doi: 10.1371/journal.pone.0213206 (PMC6402760; doi:10.1371/journal.pone.0213206)
Supplement: S2 File — (ZIP) [file pone.0213206.s002.zip › S2 File/5UTR sequences.docx]

>gi|195976767|ref|NM_001647.3| Homo sapiens apolipoprotein D (APOD), 5UTR

1 tctctctcgc acacataccc acacacacac acacacacac acacgcgcgc gcgaaaacaa

61 tatctcattt cttcttcagg gagcagctgt gaaggaaatc gggggaggag gatggacaca

121 acatcccatc tttgtgtttc gatacagact aagcttttag gccaaccctc ctgactggat

181 gggggcggcg ggcgtggcat gcatgaaaag taaacatcag agacctgaag aagcttataa

241 aatagcttgg gagaggccag tcaccaagac aggcatctca aatcggctga ttctgcatct

301 ggaaactgcc ttcatcttga aagaaaagct ccaggtccct tctccagcca cccagcccca

361 ag

>gi|725563932|ref|XM_003926188.2| PREDICTED: Saimiri boliviensis boliviensis apolipoprotein D (APOD), transcript variant X1, 5UTR

1 acacacacac acagacacac acgaaaacaa tatctcattt cttcttcagg gagcagctgt

61 gaaggaaatt gggggaggag gatggacaca agatcccatc tttgtgtttg tgttttatac

121 agactaagct tttaggccag ccctccaggc tgtaggaggc gtggcacccg tgaaaagtaa

181 acatcaaaga cccaatgaag cttataaaat agcttgggag aggccagtca tcaagacagg

241 catctcaaat cggatgattt tgcacgtgga tactgccttc atcttgatag aaaaggtccc

301 ttctccagcc acccagcccc aag

>gi|829842649|ref|XM_012780294.1| PREDICTED: Microcebus murinus apolipoprotein D (APOD), 5UTR

1 atatctcgtt tcttcttcag ggagcagctg tgaaggaaat tgggggagga gaatggacac

61 aacatcccat ctttgtgttt ggtacagagc tgagctttta ggccagcctt cctgaactgt

121 aggggtgtgg catgcatgaa aattaaacat caatgacctg atgaagctta taaaatagct

181 tgggaaaaga cagccaccaa gacaggcacc tcaaattgga agattttgca tgtggacact

241 gtcttcacct tgattttaaa acaaaggtcc cctcgacagc cacccggcct gag

>gi|672424480|ref|NM_001301354| Mus musculus apolipoprotein D (Apod), transcript variant 3, 5UTR

1 acaaacaata tctcattgtt tcttcctcag ggagcagctg tgaaggaaac taagtgaggg

61 gacagacaca gcatcccatc tttgtgcccc acacagagct cagctttgag gccagtttcc

121 cccaagctgt ggtgggtgtg gcatgcctga ctatcaaagg gcaagggcct gatgggagcc

181 tataaagtga cttgggagaa gccacacacc tcacttggag gattctgggt ggaaacttca

241 gtcatctgat ctgagaggcc tctcctgcag ccaccccacc ccaag

>gi|759101111|ref|XM_011381207.1| PREDICTED: Pteropus vampyrus apolipoprotein D (APOD), 5UTR

1 gagatggaca aaacattcca tctttgtgtt ttatacagag ctaagctttt aggtcagcct

61 tcctgaactg taggggtgtg gcatgcataa aattgaacat caatgacctg atgaagctta

121 taaaacaact tgggagaagc caaccaccaa agacaaggca tctgaagtcg gatgattttg

181 catgtggaaa ctgcctacgt cttgactaaa aaaaggttcc atctccagcc acccagccac

241 caag

>gi|671031519|ref|XM_008708344.1| PREDICTED: Ursus maritimus apolipoprotein D (APOD), 5UTR

1 ttgtgtttgg tacagagcta agcttttagg ccagccttcc tgaacgggag ggggtgtggc

61 atgcattaaa attgaacatc aatgacccaa tgaagcttat aaaatagctt gggagaagcc

121 agtcaacaag agaaggcacc ccaaattgga tgagtttgca tgtgggaact gccttcatct

181 tgacaaaaaa ggttccatcc aagccccaag

>gi|1191828899|ref|XM_001926063.7| PREDICTED: Sus scrofa apolipoprotein D (APOD), transcript variant X1, 5UTR

1 acatcccatc tttgtgtttc atacagagct cagcttttag gccagccttc ctgaactgta

61 gggggtgtgg catgcattaa aattaaacat cagtgaccct gaggaagctt ataaaatagc

121 ttaggagaag ccagtcacca acagaaggca tctcaccttg gatgattctg catgtggaaa

181 cttctttcat cttgatttaa aaaaggtcca tctccagcca cccagcccca ag

>gi|829985697|ref|XM_012729573.1| PREDICTED: Condylura cristata apolipoprotein D (APOD), 5UTR

1 gggaaaaaca gtcaccaaga caaggtatct gcaattggag gattttgcat gtgaaaattg

61 tttcatcttg atttttttaa aaaaggtagg caacccagcc ccaag

>gi|488548963|ref|XM_004465905.1| PREDICTED: Dasypus novemcinctus apolipoprotein D (APOD), transcript variant X1, 5UTR

1 gccagccaca gagacggaca tctcaaattt tggatgactt tgcatgtgga aactgccttc

61 atcttgataa aaaggttcca tctccaagcg cccagcccga g

>gi|1021087279|ref|NM_001323517.1| Homo sapiens retinol binding protein 4 (RBP4), transcript variant 2, 5UTR

1 ggcggccgct ggcacgagtg cagggtaact gagccagggc cgctggcgca tttggcctgg

61 ccgaggccac cccgcgcggc cgctccactg tgcccgaggc tgtcctggag gtgaggccgg

121 cccacaggga ccctgcccgt gcccgggctc cgggcggatt cctgggcaag

>gi|725554968|ref| XM_003922382.2| PREDICTED: Saimiri boliviensis boliviensis retinol binding protein 4, plasma (RBP4), 5UTR

1 gcagctgggc ggtcgcagcg cgctcgcctc cctagctcca cgcgcgcccg gacgcggcgg

61 ccaggcttgc acgctgctct cctcctagtg ggcggactcc tgggcaag

>gi|226958684|ref|NM_011255.3| Mus musculus retinol binding protein 4, plasma (Rbp4), transcript variant 2, 5UTR

1 gacggccgct cggctccgtc gctccacgcg cgcgcgaacg cggcggccag gcttgcacgc

61 ggctcctgct gggcagactc cggggtgag

>gi|837792831|ref|XM_004580058.2| PREDICTED: Ochotona princeps retinol binding protein 4, plasma (RBP4), 5UTR

1 cctcgctcca cgcgcgcccg cacgcggcgg ccaggcttgc gcgcgtctcc ccgtggacgg

61 actcctgggc aag

>gi|759124014|ref|XM_011361551.1| PREDICTED: Pteropus vampyrus retinol binding protein 4, plasma (RBP4), transcript variant X1, 5UTR

1 ccggacgccc cggccaggtc tgcgcgcgtc tcccggtggt gagcggcgcc cgcgcgggga

61 agggtaggga ctggcgctgt gggggcgacc gcagcgggcc gagtggtcct gcggccccgc

121 gctcacaacg cgcgtttccc gcagggcgga ttcctgggcg ag

>gi|1239969119|ref| XM_534969.6| PREDICTED: Canis lupus familiaris retinol binding protein 4 (RBP4), 5UTR

1 gtgctggccg ccggccggcg cctcccctcg gtctttcacc ccggccgtta cgaaagcgag

61 accccctccc gccccggcta taaagcgggc ggcccggcgg ccccggcgga tcgccgcctt

121 ccctggctcc acgcgcgccc cgcaccgcgg ccaggcttgc gcgcagctcc ggtgggcgga

181 ctcccgggca cg

>gi|47522929|ref|NM_214057.1| Sus scrofa retinol binding protein 4 (RBP4), 5UTR

1 gcgcgcctgg acccgcggcc acgcttgcgc gcagctcccg acgggcggac tccggagcaa

61 g

>gi|1016604691|ref|XM_016185432.1| PREDICTED: Erinaceus europaeus retinol binding protein 4 (RBP4), transcript variant X3, 5UTR

1 cgtggcgcgc tcgccccgct gctccccgcg cgcccggact gtgcagtgtt gacttctccc

61 cggcaggcag g

>gi|821102309|ref|XM_012521270.1| PREDICTED: Dasypus novemcinctus retinol binding protein 4, plasma (RBP4), transcript variant X3, 5UTR

1 gtaagtgggt gccgcggcca ccccaggccg cagctggcga ggccgctgca accgctgggt

61 aaccgggcca ggccttggcc ccggggctcg tcgccgctgg tgttttggcc ccaccgaagc

121 cacccggcgc gggagcacct ccaagccagg gggcctcccg aggcagcggg cggtatcccg

181 ggcagg

>gi|22091451|ref|NM_019101.2| Homo sapiens apolipoprotein M (APOM), transcript variant 1, 5UTR

1 agagtggact gagcagccag taggggagag agcagttaag gcacacagag caccagctcc

61 ctcctgcctg aag

>gi|831224796|ref|XM_003789008.2| PREDICTED: Otolemur garnettii apolipoprotein M (APOM), 5UTR

1 aggcaaggga actggaagca gagtggactg agcagctcta ggggagacag catttaaggc

61 accagttacc ccacacagag caccagctcc ctcctgcctg aag

>gi|9055161|ref|NM_018816.1| Mus musculus apolipoprotein M (Apom), 5UTR

1 aactgcagag caccaactct catcccgag

>gi|671038663|ref|XM_008711904.1| PREDICTED: Ursus maritimus apolipoprotein M (APOM), transcript variant X4, 5UTR

1 aaggctgtgg acagttgtgt gcctcttggg agtttgggct ctggggattg tgccacatga

61 atctatgtta aagtcttggc cggtagagaa tttgaaaggc ggcactgtgc tggcctccta

121 aaattctgtc ttctcactgg ccctcgcggc gctcctcctt ccgcgcctcc ggaagtcatc

181 ctttatttag gcggttggag gtggttctct aggtgctcgt gcgtgccgtc gcgtcgttgg

241 gactgacctc ggtgctgccg ttcccagagc cacacctggg caggtggtac tttatcgcag

>gi|95772095|ref|NM_001040640.1| Sus scrofa apolipoprotein M (APOM), 5UTR

1 gttaaggcac cagccactcc acgcggagca ccagttccct cttgcctgaa g

>gi|1016706066|ref|XM_007537146.2| PREDICTED: Erinaceus europaeus apolipoprotein M (APOM), 5UTR

1 agcagtcagt gaggaagaga gcagttaagg caccagccac tccacagagg accagctcct

61 tcctgctcta ag

>gi|471418611|ref|XM_004390745.1| PREDICTED: Trichechus manatus latirostris apolipoprotein M (LOC101344602), 5UTR

1 gactgagcag ccagcagggg acagagcagt taaggcacca gccaccccaa gcagggcatc

61 ggatctttcc tgcctgaag

>gi|586490399|ref|XM_006875603.1| PREDICTED: Chrysochloris asiatica apolipoprotein M (APOM), 5UTR

1 agagtgactg agtagccagc aggggagagg ttaaggcatc agccacccca cacagggcat

61 tagcttcctc ctgtctgaag

>gi|821123907|ref|XM_004467857.2| PREDICTED: Dasypus novemcinctus apolipoprotein M (APOM), 5UTR

1 agcaggggag agagcagttc aggcaccagc caccccacac agggcaccag ctccctcctg

61 cctgaag

>gi|38505192|ref|NM_000954.5| Homo sapiens prostaglandin D2 synthase (PTGDS), 5UTR

1 gctcctcctg cacacctccc tcgctctccc acaccactgg caccaggccc cggacacccg

61 ctctgctgca ggaga

>gi|725600796|ref|XM_010352127.1| PREDICTED: Saimiri boliviensis boliviensis prostaglandin D2 synthase 21kDa (brain) (PTGDS), transcript variant X1,5UTR

1 tgcatcacga gtcttcagtg ggctgtcctg gggccctggg tgaggccagg cctggcccat

61 aaataggggt ctcctcggtg ctctccactc ctgctgcaca cctcccttgc tcgcccacac

121 cgctggcacc gggcctcaga catctgctct gcttccagag a

>gi|1149752770|ref|XM_012784524.2| PREDICTED: Microcebus murinus prostaglandin D2 synthase (PTGDS), transcript variant X2, 5UTR

1 tctcctcagg gccctccacg cctcccgcac acctcctgtg ctcggcccac accactggca

61 ccaggccccg gacacctgct ccgctccagg agg

>gi|667282293|ref|XM_008576498.1| PREDICTED: Galeopterus variegatus prostaglandin D2 synthase 21kDa (brain) (PTGDS), 5UTR

1 tctcctcagc gtcctccgtt cctcctgccc acctcccttg ctgtgcccgc accactggca

61 gtaggcccca gacacctgct ctactccagg aga

>gi|119226207|ref|NM_008963.2| Mus musculus prostaglandin D2 synthase (brain) (Ptgds), 5UTR

1 ggctccttct gcccagtttt ccttgctttg tccacattgc tggcatcagg cccaggcacc

61 tgctctgctc tgagcaa

>gi|759148550|ref|XM_011369965.1| PREDICTED: Pteropus vampyrus prostaglandin D2 synthase 21kDa (brain) (PTGDS), 5UTR

1 cctgcctcac tctgcctgca ccgctggcac caggccccag acgcccgctc ggggaga

>gi|671023465|ref|XM_008704338.1| PREDICTED: Ursus maritimus prostaglandin D2 synthase 21kDa (brain) (PTGDS), 5UTR

1 cctcctgccg gcctccctcc ctctgcctgc actgctggca tcacaggccc tggtcacaca

61 ctcagctcca ggaca

>gi|47523539|ref|NM_214228.1| Sus scrofa prostaglandin D2 synthase (PTGDS), 5UTR

1 ctcctgcccg ccttgctcac tctgcctgca ctgccactcc gctctgggag a

>gi|1016638484|ref|XM_016187455.1| PREDICTED: Erinaceus europaeus prostaglandin D2 synthase (PTGDS), 5UTR

1 ataggggtct ccacagctgc ctctgctcct cctgcctgac tgcctccctc tgcttgcgca

61 gcctcgggac a

>gi|357933613|ref|NM_002297.3| Homo sapiens lipocalin 1 (LCN1), transcript variant 1, 5UTR

1 acagcctctc ccagccccag caagcgacct gtcaggcggc cgtggactca gactccggag

>gi|725600949|ref|XM_003941374.2| PREDICTED: Saimiri boliviensis boliviensis lipocalin 1 (LCN1), 5UTR

1 tttacatcca ggaagggatt ggtcagtcac ctgtgccagg tacccagggg accctgagcc

61 cagcggtata aagggcagcc acgggagaag cagcacagcc tctcccagcc ccagcaagcc

121 acctgtcagg ctgccgtgaa ctcagacccc ggag

>gi|1149753366|ref|XM_020289604.1| PREDICTED: Microcebus murinus lipocalin-1 (LOC105880747), 5UTR

1 tcaacgtcca acaataatga cattttacct gctccgttgc agagctgcca cggttcacgc

61 ccaggaaggc gccggtcagt cccgggagcc aagtgccctg gggcacacgc ctgtagcggc

121 ataaagggcc gccgtgcggg agccggcaca gcctctccga gctgcagcga gtgtcccgtc

181 ggtggccgtg agcgcagacc ccagag

>gi|562870831|ref|XM_006163336.1| PREDICTED: Tupaia chinensis lipocalin-1 (LOC102489459), 5UTR

1 taaagcgcag tggtttgagg ggccagccag cgagtgacct gtcgggggcc gcgagtgtag

61 acccggag

>gi|12621113|ref|NM_022945.1| Rattus norvegicus lipocalin 1 (Lcn1), 5UTR

1 gtctcctcag tctgccaagt gatctgctgt tcacctgtga gcacagactc tggag

>gi|1131163414|ref|XM_019971173.1| PREDICTED: Bos indicus lipocalin 1 (LCN1), transcript variant X1, 5UTR

1 gggccgctgc ctgagtggcc ggcacagcct ctctgcaccc agcgagtgac ctgtcggagg

61 ccgtgcrtgc agatcccagg ag

>gi|1187582851|ref|XM_020888465.1| PREDICTED: Odocoileus virginianus texanus lipocalin 1 (LCN1), transcript variant X1, 5UTR

1 agcgagtgac ctgttagcgg ctgtgcgtgc agaccccagg ag

>gi|1191803969|ref|XM_021082121.1| PREDICTED: Sus scrofa odorant binding protein 2B (OBP2B), transcript variant X1, 5UTR

1 cagcgatccg gagcccggga gcggcctgtc ctgtggttgt gagcccagac cctggatgat

61 gagggcctgc tcctggccat tggcctcggc ctcgttgctg ccctgcaggc ccaggagttc

121 ccggccgtgg ggcagccgct gcaggatctg tggggagatg gtatctgaag gcc

>gi|821115649|ref|XM_012526181.1| PREDICTED: Dasypus novemcinctus lipocalin 1 (LCN1), transcript variant X3, 5UTR

1 agaagctgct gcagcccctg agccccggca agcgtggacc ctggag

>gi|166197659|ref|NM_000606.2| Homo sapiens complement C8 gamma chain (C8G), 5UTR

1 agagtagact ctgtcctggg acttggtggt gctacccttg gcctcccaca gtcctgccac

61 cctgctgccg ccacc

>gi|725600813|ref|XM_003941350.2| PREDICTED: Saimiri boliviensis boliviensis complement component 8, gamma polypeptide (C8G), 5UTR

1 ctgctgggaa gtgcagatcg aagctgggct cacgagaagg ctgggtgtgg ggtgtggggc

61 tggcctgggg caggggttgg agagtcaagg gacggacatg gactttctgg gctgggctct

121 gtgactgcac ggcagactct ggccttggac ttggtgctgc catttccctc ccacggtctt

181 gccaccctgc cactgccacc

>gi|1327807836|ref|XM_012808283.2| PREDICTED: Otolemur garnettii complement C8 gamma chain (C8G), transcript variant X1, 5UTR

1 ccggccctca gcctccccca gtccagctgc cactgccact gctgcc

>gi|422010918|ref|NM_027062.2| Mus musculus complement component 8, gamma polypeptide (C8g), transcript variant 1, 5UTR

1 cagttattgc atgcataggg ttgagcactg accacaagtg gaaatagggg tgcagaggtc

61 aactcttaac tttgcacttg ggacacaatg cccagctcct atcaaggcta ggttctagga

121 tggaccctgg cttgggttag gggttcagta atcggaagac agatgtggac ttcctggact

181 gtgcttcctg tggcactgga cagtggactc tgacctagga cagtgcaccc ctccacctcg

241 ctgtcctgtc acctactgtc cgtagcaggt tggcc

>gi|1124007216|ref|XM_019756732.1| PREDICTED: Rhinolophus sinicus complement C8 gamma chain (C8G), transcript variant X3, 5UTR

1 ctgcgcgctg gggctcaccc ggaagagcgc ccctggcggc cgggaggagc ggcggtgcgc

61 cagggtccgt gggcggagct gcgggcaaga cgctggggcg gtgggttcga gacccagttc

121 tggcgctccc gttgtctggc cccaaacaag cggcttcagg agcctcgtgt ttttggctgt

181 gggaataaaa ccagcgttta cctcaaggag ctcttggcgt tttaaaggat ataagtgtac

241 acagggctgg gcactggcca cagtggaaat tcgctggggg ctcctgcaaa aatagccaga

301 ggtcaactcg gactttgtac acagggaatg gcgccagggc cctgctggga agtgcagagt

361 cgaggtcaga tttccagggg ctcggtcgtc ctgcccttgg ttgccccagt cccgtcactg

421 tcactgccac cgcc

>gi|671023350|ref|XM_008704275.1| PREDICTED: Ursus maritimus complement component 8, gamma polypeptide (C8G), 5UTR

1 ggctggtctg agggcaaggc tcactcacct cgtgagttcg cgaccccgcc ccgccgctcc

61 cagctgctgg ggccctgggt tagggggtcg tggacgagga accagaggtc aacccggatt

121 ttgtaagcag gatatggcgc cagggccctg ccgggaagtg cagagtcgag gaggctgggg

181 gtggactctt gtggggggca gggtgcttgg tgaggcagga ggaccaatgg ggacttcctg

241 ggctggaggt ggacgggcgc cgacttcctg tgccaggctt ctgggcggca ctggacagtg

301 gactctgccc caggacttgg ccgtcctgcc ctggcctccc ccagtcccag cactgttact

361 gccaccacc

>gi|148223226|ref|NM_001097452.1| Sus scrofa complement C8 gamma chain (C8G), 5UTR

1 atcgtcctgc tctctgtctc caccggtcct gtcgccgttg ccgccgtggc c

>gi|1016638451|ref|XM_016187449.1| PREDICTED: Erinaceus europaeus complement component 8, gamma polypeptide (C8G), 5UTR

1 ggacagtgga cttctgaccc agggactggc tgccctgacc tcgggctctt cctgtctcag

61 cggcactgtc actgccacc

>gi|1328813993|ref|XM_023542194.1| PREDICTED: Loxodonta africana complement C8 gamma chain (C8G), 5UTR

1 ggagggtgct gggcagggca ccttcacgag cccggtttcc ttggctgagg ataagaccag

61 ttcctacctg cagtcctgtt ggtggcttaa gggaaataaa tgtttaaagg gctgagcgac

121 ggccaccagt gggaagtgga gggtccaggt gtgcatggac aaagggcccg aaatcaactc

181 ggagtttgta ggcaggaagt gcaaatcaag gtcagacgtc cagggcaagg ccggggctca

241 caggagactg gtggaccctg gcccagcaag gggatgtggg taggcggagg ccagcctgga

301 cttcccagct gggcctgacc cctcccccag gctgggctcc tgggtggcac tga

>gi|930697465|ref|NM_005564.4| Homo sapiens lipocalin 2 (LCN2), 5UTR

1 agggccaccc aggtgagcct ctcactcgcc acctcctctt ccacccctgc caggcccagc

61 agccaccaca gcgcctgctt cctcggccct gaaatc

>gi|831218485|ref|XM_003785429.2| PREDICTED: Otolemur garnettii lipocalin 2 (LCN2), 5UTR

1 cattctcctc ctccttcacc tctgccagtc agcagctgcc cagagtgcct gcttcctccg

61 ccctgaaacc

>gi|667244376|ref|XM_008579050.1| PREDICTED: Galeopterus variegatus lipocalin 2 (LCN2), transcript variant X2, 5UTR

1 gccaggctct tcactcacct cctccatctg cctggcccag cagctgcccg agctcccgtt

61 tcctcagccc tgaaatc

>gi|34328048|ref|NM_008491.1| Mus musculus lipocalin 2 (Lcn2), 5UTR

1 agacctagta gctgtggaaa cc

>gi|671012212|ref|XM_008698716.1| PREDICTED: Ursus maritimus lipocalin 2 (LCN2), 5UTR

1 cactagcttc tcttcagctg ctgtctggca cgccgagttt cctaaggcct gaagtc

>gi|1048450748|ref|XM_017641830.1| PREDICTED: Manis javanica lipocalin 2 (LCN2), 5UTR

1 gcggcctcca cacttgcccc tccttcagct gctgcctgga gcttgtcagc ccagaatcc

>gi|346986373|ref|NM_001244410.1| Sus scrofa lipocalin 2 (LCN2), 5UTR

1 tcttgccaag tatttcagca ggagtgctgg caattgcctc acccttcctg gatttggcaa

61 aatatgattc actccaccct ggggtgggcc actggtcagg tggacggacc ataaataggg

121 tcccccgggc agccttctca ctcgcctccc cctccttcag cggctgtccg gaggaccaga

181 ctcctcggcc ctgcaacc

>gi|1016671166|ref|XM_016190699.1| PREDICTED: Erinaceus europaeus lipocalin 2 (LCN2), 5UTR

1 ccataaatag ggtccccccc aggctgggct cctcacttgc ctcttcttcc ttcaccatct

61 gagtgccagc tccctcagtc ctgaaacc

>gi|821105921|ref|XM_012523133.1| PREDICTED: Dasypus novemcinctus lipocalin 2 (LCN2), 5UTR

1 ccacccagag ctgggttggc agacaggtgg gcccataaat agagtcccgc aggcgaaccc

61 ctcacttgcc tcctccacca cccgccaggc ccagcatccg cctcgagctc cggcttcctc

121 agccctgcaa tc

>gi|62988356|ref|NM_178469.3| Homo sapiens lipocalin 8 (LCN8), transcript variant 2, 5UTR

1 gctcctcttc ctctcaggct ccgagacggc cccagcagcg tccaccgctg tccatatgcc

61 aggagggtgg ctgggcaggc tgctgtctag gccaggctaa cccctgcggt gggcgtgggt

121 gtcaccaggg ccgatggcgc ttgtgcagaa acccacgtct ctgagctgcc agcagccaag

181 ctgtcctgat gacattcccg ggtgggcgca caagcctgca ctgtccgtat agaatcggcc

241 caggctgtgc agcaggggaa cccggagccc ggaccccgcc acggaggcca ggctgccgtg

301 caccatcctg ggtgtcctcg tggtgctccg ggcgcaggtg gcagcagcc

>gi|820997544|ref|XM_003279787.3| PREDICTED: Nomascus leucogenys lipocalin 8 (LCN8), 5UTR

1 tctcaggccc ccagacggcc ccagcagcgt ccaccgctgt ccacacatca ggagggtgac

61 cgggcaggct gctgtctagg ccaggctaac ccccgcggtg ggcgtgggtg tcaccagggc

121 cgg

>gi|1149742755|ref|XM_012784405.2| PREDICTED: Microcebus murinus lipocalin 8 (LCN8), 5UTR

1 tccccggcga ccgcgctctc tccccaaata aaagcccggg cgctgcagga gcggagctgc

61 ctgcgggagc ccagaccccg cc

>gi|14994307|ref|NM_033145.1| Mus musculus lipocalin 8 (Lcn8), 5UTR

1 aagccacaag ccaggccctg ag

>gi|1196067892|ref|XM_005083766.2| PREDICTED: Mesocricetus auratus lipocalin 8 (Lcn8), 5UTR

1 ccctccataa acagaagcca caagacaggc cctgag

>gi|1304944854|ref|XM_023243073.1| PREDICTED: Felis catus lipocalin 8 (LCN8), 5UTR

1 ccaaggctga tctgaaagag ccagaaagag aaagctgttc tgagcccatg gggcctgggc

61 ccggcatgcc cctcgggctt ggggctgtgg tcccgagccg ccccatgggg gtggggggcc

121 tcctccctcc tcctctcggg gaccccaggc gggcccaccg tggggccgtg ggtgtccccg

181 ggagcttgtg caacaccgtc ccgatataaa cccaggtgct ggggcagcag aggctccgtg

241 agcggcg

>gi|671023344|ref|XM_008704272.1| PREDICTED: Ursus maritimus lipocalin 8 (LCN8), 5UTR

1 tcccggccag ctcagcctct tgcggactgt cttccttctc acag

>gi|1246175866|ref|XM_022577953.1| PREDICTED: Delphinapterus leucas lipocalin 8 (LCN8), transcript variant X1, 5UTR

1 aaacccaggt gcggtgggca caggtgcggg gagcatagac cctcc

>gi|965898818|ref|XM_015089717.1| PREDICTED: Ovis aries musimon lipocalin 8 (LCN8), transcript variant X2, 5UTR

1 gtgcatgagg gtggagggtg atgggttggc agcaataagc tggtagaact gaaaaaagta

61 aaaactaaga gcttcagagg gaaggtccag gccccagggc tggtttccaa ggcggctgct

121 cggtcctcca gcatggccgt gcagaaccag gcctccccca cggggcaggg ctgctggcca

181 ccctcctcag ggggccttca cgtccagctg gcccgctccc tgccccgtct ctcgggctct

241 gaccaccgct gcctctgctc aggggcaggc atcacaaggc caggtctcct tggagacagg

301 gcaccagccc tggggcagct gcaaacaggc ccttgtgggg acccagggcc cccacgggga

361 cccgcggaag gcccgccgcc tgctgcttcg tctgccggct ccctgcctgg gaggtctagg

421 gcgaggggtc ggcctccccg ggtccggttc ggctgcccga atgggcttgg ctccggtcct

481 ttctctag

>gi|1034664955|ref|XM_011518561.2| PREDICTED: Homo sapiens lipocalin 12 (LCN12), transcript variant X1, 5UTR

1 cctgaggaga gcccacacca agcctgccct gggcaccacc tctcctgcca gtcctgagga

61 gggaggcaga ggggcacacc cccttgggag gggcacctgc tgggt

>gi|725600807|ref|XM_010352131.1| PREDICTED: Saimiri boliviensis boliviensis lipocalin 12 (LCN12), transcript variant X5, 5UTR

1 caacgggatg cagggcctca ggaacgagaa gacgaggtgg aaggtggccc tgagggggcc

61 ctcctaccct gcctgactgc cccgcccagc tcaccccagg cctgccctgg gcaccacctc

121 tcctgccagg cctcaggagg gaggcaggag gtgcatcccc ttgggagtgg cacctgcagg

181 gtacacacat aaagaggtgg gtctctgggt catcctgccc atagccactt ccttttcgct

241 gtccctgtgg gcctggcagc tgccagg

>gi|1149752730|ref|XM_012784525.2| PREDICTED: Microcebus murinus lipocalin 12 (LCN12), 5UTR

1 atttaattgt tagcaatctt gcctgagacc cacaggcatc aggggacctg ggggggtctc

61 agggtctcat gagcctgggg gtcccgcctg cctcgtgggg aaggcagggt gctgctgtgc

121 ggaaagtgcc tgcagggggg cggcaggact cac

>gi|755498040|ref|XM_011239211.1| PREDICTED: Mus musculus lipocalin 12 (Lcn12), transcript variant X1, 5UTR

1 ttttccttgg ctaggaatca agagaccctg gcaagtgttt aggatctgag agggtcagcc

61 ctgccctgtt tctacaccca cctgtctgct gtaaccctca gggccctggc cacgtaggct

121 gtgaagaacc aggtttctga gcaagcttca ccagcagccg ctcctcgggc aggctgtgtc

181 cttctggagc ctggtggctg ttggg

>gi|1124007155|ref|XM_019756702.1| PREDICTED: Rhinolophus sinicus lipocalin 12 (LCN12), transcript variant X1, 5UTR

1 tgccacccct gcctctccat ttcctgccca ggtgcccccc atgtttggtg gacaggatgg

61 tggagggcct ccaagcctat gcgcccacct ctcctcctgc caggaggatg gtggagcggg

121 cacaccccag ggaggggcgt cagtgagggg ggaggacggg cccataaaga ggtgagtcag

181 cagcatcaca ggccacttcg tcccttggtg ccgcagggat ggggcccctg tgtgccctgg

241 gggtgctgct caccctgttg gaagctggga agggccagac cctgaagccc cctccagcca

301 tcaccccggt cttgcagagt ttccaagagg accaggtctg gggctggaag ggagggggag

361 gcagccagct gtaggcacgg ccccggagtc cagtaccacc agccctgcac tgaccagggg

421 gcccagccct gctctcagcc accctcctgc catggaaacc cctgacctcc aacagaaaag

481 gacccttgct aggtcagcat tccatcctgt aacccctgtc aatccctgtg aggggaggac

541 accgcagtcc ctctgcacag acagggaggc taaggctcag agaggccagc tgctggccag

601 ggtcaccccg tcctgccaga cccagggcag cccctggctc cggccagcac cactggtgca

661 ggcccagcct gggaacctgg tgaggggtct tgtggccatc ggggctctgc ccatccccct

721 cccctgcagt tccaggggga gtggttcgtc gttggcctgg cgggcagcac atatggacgg

781 gcagacaggt catttctgaa ctctttcatt gcaacgtttg agcaaactgg aaaccgccgc

841 ctcagagcat cctatgcc

>gi|671023348|ref|XM_008704274.1| PREDICTED: Ursus maritimus lipocalin 12 (LCN12), 5UTR

1 ccccccgccg gttcccgcag ttccaggggg aatggtttgt tgtcggccta gcgggcagca

61 cccacagcaa gacggacaga tttctgttga accccttcat tgcaacgttc gagcaaaatg

121 gaaacagccg ccttgaagtg tcgtatgcc

>gi|1187582866|ref|XM_020888474.1| PREDICTED: Odocoileus virginianus texanus lipocalin 12 (LCN12), transcript variant X1, 5UTR

1 ctggggttgg cagcacggct gcatcccgcc ccgtccctgc tggg

>gi|830000087|ref|XM_012721162.1| PREDICTED: Condylura cristata lipocalin 12 (LCN12), 5UTR

1 tgcccgcgcc ggccccgccc cagacccccg ccggggtggc agggtctgag gagggtgggg

61 cagccagccc agccatgccc tcagctcaag gaggccacgg gggccagccg ggggagccca

121 taaagagggc gcctctgggc ctgcacggtc agcagcccac tccctggcga g

>gi|488547532|ref|XM_004465216.1| PREDICTED: Dasypus novemcinctus lipocalin 12 (LCN12), 5UTR

1 ccctaaccac tgggccaaag tccgtttccc atccgagccc ttctgagtgg tagaggaccc

61 tgggcctgtg tcccgaggag ccggcggccc ccaggggcct cccgatacag agcccgcaca

121 gaccgccagt gcccaagcca ggctccgagc agcatgcgca gaagccggag gcaacggggc

181 ccggctctgc tggatcacag cttcggaggt tgagggccgc ggggttccag gcacagctgg

241 acccaggggt tcggccgctg tgatcaggcc caggccctgc tgtgtccggg caggccctcg

301 cctcgtggcc gcgagacagc aaggggcacc ctgcgccccc atcatccccg ggccccgtgc

361 cagagccggg gagcgcccgg gcaagcctgg gaatgggctc tgagcggccc ttccgtcgtg

421 tgcccttgct ggacgagtcc ccgtgctcgg ggtgccctct gatccgccag cccggatgcg

481 gcgcccgctc tctgcttgag gaaggggccg cgcagggcct ccagggtact tcttgcaaac

541 ctgcggtgag cacagtcgcc tgcgcctgct ggagtcaccg gtgcaggctc gaccaagccc

601 agaaccccct ccctggggac acctggttcc tgggaagacc caacttgccc agcttggggc

661 aggggcttc
