# Supplementary material for: Characterization of mammalian Lipocalin UTRs in silico: Predictions for their role in post-transcriptional regulation
Source: PLoS One. 2019 Mar 6;14(3):e0213206. doi: 10.1371/journal.pone.0213206 (PMC6402760; doi:10.1371/journal.pone.0213206)
Supplement: S1 Table — (DOCX) [file pone.0213206.s006.docx]

*FPROM prediction coincidence with NNPP of higher probability (highlighted in bold in the table below).*

*GPMiner (more adjusted to mouse promoters) also show results compatible in NNPP for both Lipocalins.*

| **RBP4 human** | | | | |
| --- | --- | --- | --- | --- |
| **NNPP** | | **FPROM** | | |
| **Promoter region** | **Score** | **TSS** | **Score** | **TATAbox** |
| 893-943 | 0.87 | - | - | - |
| **973-1023** | **0.99** | 1013 | 1.487 | 983 |
| 1811-1861 | 0.83 | - | - | - |
| **2393-2443** | **1.00** | 2433 | 13.77 | 2402 |
| **Rbp4 mouse** | | | | |
| **NNPP** | | **GPMiner** | | |
| **Promoter region** | **Score** | **TATAbox** | | |
| - | - | 1734-1739 | | |
| 1752-1802 | 0.80 | 1761-1766 | | |
| 1894-1944 | 0.83 | - | | |
| **1945-1995** | **0.89** | 1954-1959 | | |
| 2011-2061 | 0.84 | - | | |
| **2487-2537** | **1.00** | 2495-2500 | | |

| **LCN2 human** | | | | |
| --- | --- | --- | --- | --- |
| **NNPP** | | **FPROM** | | |
| **Promoter region** | **Score** | **TSS** | **Score** | **TATAbox** |
| 1576-1617 | 0.88 | - | - | - |
| **1960-2010** | **0.98** | 2000 | 3.051 | 1970 |
| **Lcn2 mouse** | | | | |
| **NNPP** | | **GPMiner** | | |
| **Promoter region** | **Score** | **TATAbox** | | |
| 222-272 | 0.92 | - | | |
| 608-658 | 0.81 | - | | |
| - | - | 1196-1201 | | |
| **1930-1980** | **0.99** | 1942-1947 | | |
